# Moloney Murine Leukemia Virus-like Nanoparticles Pseudo-Typed with SARS-CoV-2 RBD for Vaccination Against COVID-19

**DOI:** 10.3390/ijms26136462

**Published:** 2025-07-04

**Authors:** Bernhard Kratzer, Pia Gattinger, Peter A. Tauber, Mirjam Schaar, Al Nasar Ahmed Sehgal, Armin Kraus, Doris Trapin, Rudolf Valenta, Winfried F. Pickl

**Affiliations:** 1 Institute of Immunology, Center for Pathophysiology, Infectiology and Immunology, Medical University of Vienna, 1090 Vienna, Austria; bernhard.kratzer@meduniwien.ac.at (B.K.);; 2 Department of Pathophysiology and Allergy Research, Center for Pathophysiology, Infectiology and Immunology, Medical University of Vienna, 1090 Vienna, Austria; pia.gattinger@meduniwien.ac.at; 3 Laboratory for Immunopathology, Department of Clinical Immunology and Allergology, I. M. Sechenov First Moscow State Medical University (Sechenov University), 119991 Moscow, Russia; 4 LIFE Improvement by Future Technologies (LIFT) Center, 115478 Moscow, Russia; 5 Center for Molecular Allergology, Karl Landsteiner University, 3500 Krems, Austria; 6 Karl Landsteiner University of Health Sciences, 3500 Krems, Austria

**Keywords:** SARS-CoV-2, COVID-19, antibody response, SARS-CoV-2 immunity, VNP

## Abstract

Virus-like nanoparticles (VNPs) based on Moloney murine leukemia virus represent a well-established platform for the expression of heterologous molecules such as cytokines, cytokine receptors, peptide MHC (pMHC) and major allergens, but their application for inducing protective anti-viral immunity has remained understudied as of yet. Here, we variably fused the wildtype SARS-CoV-2 spike, its receptor-binding domain (RBD) and nucleocapsid (NC) to the minimal CD16b-GPI anchor acceptor sequence for expression on the surface of VNP. Moreover, a CD16b-GPI-anchored single-chain version of IL-12 was tested for its adjuvanticity. VNPs expressing RBD::CD16b-GPI alone or in combination with IL-12::CD16b-GPI were used to immunize BALB/c mice intramuscularly and subsequently to investigate virus-specific humoral and cellular immune responses. CD16b-GPI-anchored viral molecules and IL-12-GPI were well-expressed on HEK-293T-producer cells and purified VNPs. After the immunization of mice with VNPs, RBD-specific antibodies were only induced with RBD-expressing VNPs, but not with empty control VNPs or VNPs solely expressing IL-12. Mice immunized with RBD VNPs produced RBD-specific IgM, IgG_2a_ and IgG_1_ after the first immunization, whereas RBD-specific IgA only appeared after a booster immunization. Protein/peptide microarray and ELISA analyses confirmed exclusive IgG reactivity with folded but not unfolded RBD and showed no specific IgG reactivity with linear RBD peptides. Notably, booster injections gradually increased long-term IgG antibody avidity as measured by ELISA. Interestingly, the final immunization with RBD–Omicron VNPs mainly enhanced preexisting RBD Wuhan Hu-1-specific antibodies. Furthermore, the induced antibodies significantly neutralized SARS-CoV-2 and specifically enhanced cellular cytotoxicity (ADCC) against RBD protein-expressing target cells. In summary, VNPs expressing viral proteins, even in the absence of adjuvants, efficiently induce functional SARS-CoV-2-specific antibodies of all three major classes, making this technology very interesting for future vaccine development and boosting strategies with low reactogenicity.

## 1. Introduction

The beta Corona virus SARS-CoV-2 triggered a worldwide pandemic, since its first description in Wuhan, China, in 2019. Until today, the pandemic has caused more than 676 million cases and more than 6.8 million deaths worldwide. The COVID-19 pandemic dramatically accelerated the development and application of new vaccines, among them mRNA- and viral-vector-based vaccines [1,2,3,4,5,6]. These vaccines contributed strongly to the anti-SARS-CoV-2 immunity present in the population today and effectively protected vaccinees from a severe disease course, although the vaccines were unable to induce (long lasting) sterile immunity and therefore could not prevent further spreading of the disease [4]. However, the death rates markedly decreased since the introduction of effective vaccination programs, the increasing immunity due to mild infections and the emergence of new virus variants, e.g., from the Omicron lineage [7]. In addition, vaccination against COVID-19 also reduces the post-acute and long-term symptoms of COVID-19 [8,9,10,11].

One way of improving the efficacy of vaccines (especially non-live, protein-based vaccines) is the use of adjuvants. Adjuvants typically consist of inorganic salts [12], oils [13], bacterial products [14] or whole virosomes [15] and variably trigger TLR- or NLR-driven danger responses; thereby, they lead to the production of cytokines, the attraction of immune cells and the facilitation of antigen-uptake and processing. Virus-like nanoparticles (VNPs) are virosomes, consisting of virus core proteins, which, due to their virus-like symmetry, repetitive structural features and size, enhance immunogenicity [16]. Upon injection, their size of about 100 nanometers, which gave them their name, facilitates their preferential transport towards the regional lymph nodes [17], and thereby helps to mount effective humoral and cellular immune responses against their immunogenic constituents [18,19]. We have shown in the past that enveloped Moloney murine leukemia virus (MoMLV)-derived VNPs can be efficiently decorated with cytokines of choice which retain their immunostimulatory and differentiating potential in vitro, and which thus can act as adjuvants locally [20,21].

In general, vaccines for the induction of immunity against SARS-CoV-2 are based on different technologies, such as attenuated SARS-CoV-2 virus, recombinant virus antigens alone or as fusion proteins with immunological carriers or the genetic information for their in vivo production [22,23] (https://www.who.int/publications/m/item/draft-landscape-of-COVID-19-candidate-vaccines, accessed on 15 April 2025). In the past, several VNP-based candidates have been investigated in clinical and pre-clinical studies [24]. So far, the most advanced clinical studies published were based on SARS-CoV-2 VNP, which either expressed full-length S-protein, RBD alone or a combination of S, NC and M proteins in the presence of AL03 or CpG DNA as adjuvants and have demonstrated the induction of protective neutralizing antibodies in human subjects in large phase I and II trials [25,26,27,28,29]. The presence of NC and M proteins in those vaccines was considered to enhance cellular immunity against SARS-CoV-2. Besides SARS-CoV-2-based VNPs, adenovirus- [30], bacteriophage lambda- [31], Qb- [32], baculovirus- [33], cucumber mosaic virus- [34], Hepatitis B surface antigen- [35], HIV-1- [36], influenza- [37], Newcastle disease- [38], rabies- [39] and rhabdovirus-based VNPs [40] and even artificially designed VNPs [41] have been investigated as potential platforms for SARS-CoV-2 vaccines. All of these variably showed the induction of protective neutralizing antibodies in pre-clinical studies. Moreover, MoMLV-based VNPs expressing full-length S-proteins fused to VSV-G have been shown in the past to induce protective immunity after one vaccination in hamsters [42], highlighting the potential of the MoMLV-based VNP platform for inducing SARS-CoV-2 immunity. The majority of these vaccines relied either on the expression of full-length spike protein [25,26,27,28,29,30,36,37,43,44,45], a stabilized pre-fusion form of it [38] or RBD [3,46,47,48,49]. In accordance with previous studies, here, we relied on the use of a functionally important subunit of the S-protein, referred to as receptor-binding domain (RBD) of the SARS-CoV-2 spike protein as immunogen, which is responsible for virus-binding to its receptor, ACE-2 [50]. Antibodies against RBD are functionally important because they inhibit the binding of the virus to its cellular receptor ACE-2 and thus confer protection against infection with SARS-CoV-2 [51,52].

RBD-based VLP vaccination approaches have been established previously by applying cucumber mosaic virus [46,47], Qb [3], cowpea mosaic virus [3], plant-expressed influenza [48] or yeast particles [49]. However, the production process for these particles did not always allow eukaryotic co-translation of core proteins, which is required for the production of VLP and RBD, which is used as a pivotal immunogen. Eukaryotic production of RBD is, however, mandatory for vaccination success, since only antibodies directed against folded RBD are able to block interaction of the viral S-protein and the cellular receptor ACE-2 and are therefore highly relevant to protect against infection with SARS-CoV-2 [52,53]. Notably, the MoMLV-based VNP platform used herein relies on the same host cell (HEK-293T) for the expression of both the (i) immunogen (RBD) and (ii) the viral core proteins (MoMLV-derived gag-pol); this represents the ‘budding principle’ which also provides the lipid-bilayer for envelopment of the VNP. Besides this advantageous feature, HEK-293T-producer cells are easily scalable for the production of large amounts of VLPs, as has been demonstrated for the production of HIV-based VLPs [54] and are, at the same time, a cost-effective production platform, as has been shown for, e.g., Hepatitis B VNPs [55]. Moreover, MoMLV-based VNPs are non-infectious; additionally, unlike other VNPs, such as rabies- or HIV-based VNPs [39,56], they are unable to induce false-positive medical diagnostic test results in vaccinees. The lipid envelope of MoMLV enables co-expression along with potential immunogens in addition to immunostimulatory molecules, like cytokines. We have shown before that IL-2-decorated VNPs enhance the function of CD8^+^ T cells [57] and IL-4- and GM-CSF-decorated VNPs, which appear as distinct particles enveloped by a lipid plasma membrane bilayer in electron microscopy [20,58,59], are able to differentiate monocytes into moDC [20]. However, anti-viral immunity is generally believed to depend on the successful induction of a strong, Th1-polarized immune response [60], governed by IFN-γ production and induced by monocyte/macrophage-derived IL-12 [61]. IL-12 is a heterodimeric cytokine, which consists of a p40 and p35 subunit linked together via disulfide bonds. Although similar concepts have already been applied for influenza-based VNPs [62], no in-depth evaluation has been performed so far into the issue of whether the repetitive application of such cytokine-enhanced VNPs will lead to the development of anti-cytokine antibodies and might have an undesirable effect. This question is highly relevant, since the emergence of new virus variants requires repetitive booster vaccinations.

Accordingly, we here investigated whether SARS-CoV-2 S, RBD and NC proteins can be expressed as GPI-anchored molecules on the surface of MoMLV VNPs and whether such decorated VNPs would, firstly, be recognized by antibodies and T cells of convalescent patients and, secondly, be able to induce humoral and cellular immune responses in mice; it is also important to determine whether such anti-viral responses would be enhanced by co-expressed IL-12.

The efficient expression of SARS-CoV-2 molecules on HEK-293T-producer cells and VNPs was confirmed by their recognition with antibodies and T cells of convalescent patients. Balb/c mice immunized with RBD-expressing VNPs in the absence of IL-12 produced RBD-specific IgM, IgG_2a_ and IgG_1_ after the first immunization; while, RBD-specific IgA appeared only after a booster immunization. The additional presence of IL-12-GPI led to later and lower occurrence of RBD-specific antibodies and the induction of anti-IL-12 autoantibodies. The VNP-induced antibodies reacted exclusively with folded but not with unfolded RBD, neutralized SARS-CoV-2 virus and enhanced cellular cytotoxicity (ADCC) against RBD-protein-expressing targets but not against control cells. Notably, booster injections progressively increased IgG antibody avidity. Interestingly, the last immunization with RBD–Omicron VNPs mainly enhanced preexisting RBDHu-1 Wuhan-specific antibodies.

## 2. Results

### 2.1. SARS-CoV-2 Antigens Are Expressed on the Surface of HEK-293T Cells

**Figure 1:**
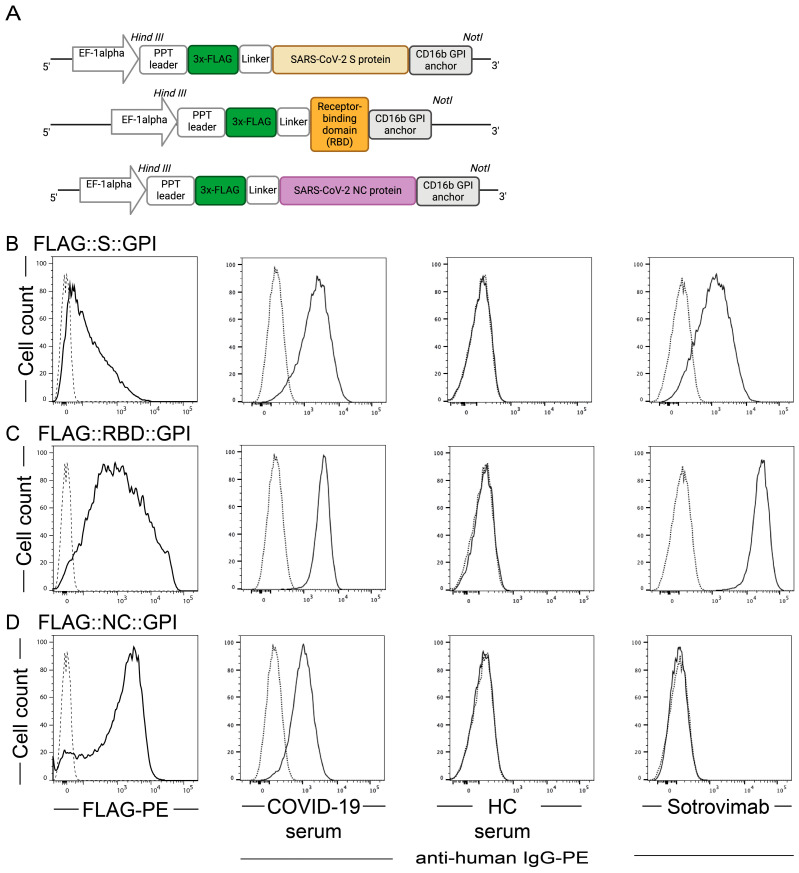
Schematic depiction of expression cassettes and expression of SARS-CoV-2 proteins by HEK-293T VNP producer cells, as detected by an anti-FLAG-tag antibody, anti-RBD antibody Sotrovimab and human sera. (**A**) Schematic representation of the SARS-CoV-2 proteins in the expression vector pEAK12. Lower panels show the HEK-293T expression of (**B**) S-protein, (**C**) RBD protein and (**D**) NC-protein, as detected by anti-FLAG mAb (FLAG-PE), by serum of a COVID-19-convalescent patient (COVID-19 serum) in comparison to the staining obtained with serum of a SARS-CoV-2 non-exposed control subject (HC) (all as solid lines) or the monoclonal antibody sotrovimab. The dotted lines represent the fluorescence obtained with control-vector-transduced HEK-293T cells using the indicated primary and secondary staining reagents, indicating the background fluorescence. X-axes show the fluorescence intensities and Y-axes show the cell numbers. Data are representative for three independent experiments performed with the sera of five COVID-19-convalescent patients and five SARS-CoV-2 non-exposed control individuals.

To analyze whether the surface and structural proteins of SARS-CoV-2 can be used to efficiently pseudo-type virus-like nanoparticles (VNPs), we modified the corresponding S- and NC-encoding sequences of wild-type SARS-CoV-2 by fusing them at their C-terminus with the glycosylphosphatidylinositol (GPI) anchor attachment sequence of CD16b, a well-described GPI-anchored molecule (Table S1). The RBD sequence of the S-protein was modified similarly. To optimize expression and allow for universal detection, expression of molecules was put under the control of the pre-pro-trypsin (PPT)-leader sequence and a 5′-terminal FLAG-tag sequence was attached followed by a linker sequence (GGGGS) (Figure 1A).

To assess the expression of the SARS-CoV-2 constructs by flow cytometry, stable transfectants were prepared as described in [63]; alternatively, the HEK-293T cells were transiently transfected [64] with pEAK12::FLAG::S::GPI, pEAK12::FLAG::RBD::GPI and pEAK12::FLAG::NC::GPI, respectively. Notably, anti-FLAG staining revealed surface expression of FLAG::S::GPI, FLAG::RBD::GPI and FLAG::NC::GPI with staining of 71.1 ± 27.0%, 89.9 ± 6.0% and 86.6 ± 2.8% of the transfected cells, respectively; whereas, only 2.8 ± 3.3% of control transfected cells (empty vector) reacted with the anti-FLAG antibody (Figure 1). Similarly, a 1:100 dilution of serum from a COVID-19 convalescent patient (B115) but not serum of a SARS-CoV-2 non-exposed individual (A001) positively stained a large fraction of FLAG::S::GPI and FLAG::RBD::GPI transfectants when counterstained with goat-anti-human IgG, while its reactivity with the FLAG::NC::GPI transfectants was moderate (Figure 1). Similarly positive results were obtained with sera of further COVID-19-convalescent and with the monoclonal, neutralizing antibody sotrovimab [65] but not with previously non-exposed individuals (*n* = 5 for each condition; Table S2), confirming the presence of neutralizing epitope recognized by sotrovimab (Figure 1).

### 2.2. Generation of VNPs Which Are Decorated with SARS-CoV-2 Antigens

**Figure 2:**
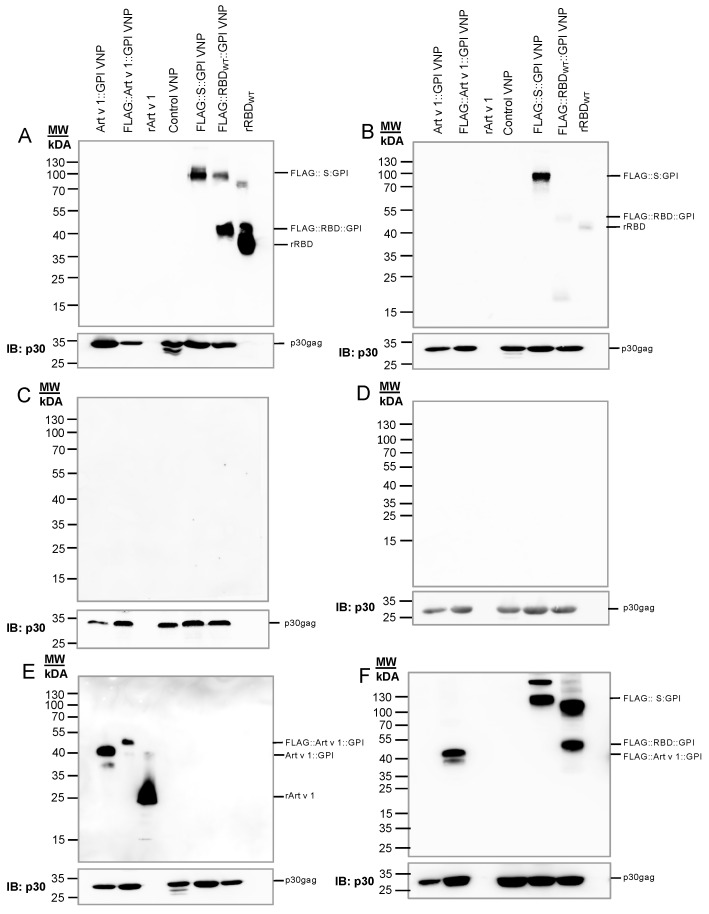
Sera of COVID-19-convalescent subjects recognizing SARS-CoV-2 proteins expressed on immunoblotted VNPs. Immunoblot analyses demonstrating the recognition of SARS-CoV-2 proteins expressed on (VNPs) by a serum pool of COVID-19-convalescent subjects or a non-SARS-CoV-2-exposed subject. Ten µg/lane of purified VNPs expressing the indicated SARS-CoV-2::GPI-anchored fusion proteins, VNPs expressing Art v 1::GPI as control for a non-viral GPI-anchored protein, empty control VNPs or 0.25 µg/lane of recombinant RBD or 0.5 µg/mL of recombinant Art v 1 were separated using 11% SDS-PAGE and transferred onto nitrocellulose membranes. The reactivity of a serum pool from five COVID-19-convalescent individuals with purified VNPs and control proteins which had been separated under (**A**) non-reducing or (**B**) reducing conditions (upper panels, respectively) or with a serum from a non-SARS-CoV-2-exposed subject under (**C**) non-reducing or (**D**) reducing conditions (upper panels, respectively). (**E**) Reactivity of an anti-Art v 1 mAb (clone 5) under reducing conditions (upper panel). (**F**) Reactivity with anti-FLAG mAb under reducing conditions. Below each blot, the reactivity of the rat-anti-p30gag mAb R187 with MoMLV core proteins is shown. The position of molecular mass markers is indicated in kilo Daltons (kDa). Control VNPs were generated by HEK-293T-producer cell transfection with OGP vector and an empty pEAK12 vector. The data shown are representative of three independent experiments.

The RBD sequence of the SARS-CoV-2 S-protein is the major target for the induction of antibody-based immunity against SARS-CoV-2 [22,52,66]; however, other conserved sequences of the S-protein might also be of importance [67]. To examine the capability of FLAG::RBD::GPI or FLAG::S::GPI to decorate (pseudo-type) VNPs, virus budding was induced in HEK-293T cells [68] transfected with SARS-CoV-2 GPI expression constructs. This was followed by the biochemical characterization of the released VNPs by immunoblotting. To this end, HEK-293T cells were transiently transfected with MoMLV gag-pol encoding the core protein and the polymerase of the Moloney murine leukemia virus (OGP) [69] in parallel with either FLAG::RBD::GPI, FLAG::S::GPI or, for control purposes, Art v 1::GPI, FLAG::Art v 1::GPI or control DNA (empty vector). After three days, the VNPs secreted into the supernatants of the HEK-293T transfectants were isolated by ultrafiltration and ultracentrifugation and analyzed to determine the presence of immunoreactive SARS-CoV-2 proteins. The VNPs were stable for at least 4 weeks without alteration of their biological activity, as has been demonstrated in previous studies [59]. For that purpose, equal amounts of VNPs were resolved by SDS-PAGE under non-reducing or reducing conditions, followed by their transfer to nitrocellulose membranes and immunoblotting with a serum pool from COVID-19-convalescent patients (Figure 2A,B) or non-SARS-CoV-2-exposed control subjects (Figure 2C,D). FLAG::RBD::GPI was detected as 40 kDa (monomer) and 95 kDa (dimer) bands by the serum pool of the COVID-19-convalescent patients, which also recognized rRBD_WT_ as a 35–40 kDa band (Figure 2A). Notably, the RBD reactivity of the convalescent serum pool disappeared when the VNP lysate was separated under reducing conditions (Figure 2B). Similarly, the rRBD-His protein used as positive control was strongly recognized by the COVID-19-convalescent serum pool when resolved under non-reducing but only weakly under reducing conditions. This was in clear contrast to the COVID-19-convalescent serum reactivity with FLAG::S::GPI protein, which was recognized under both non-reducing and reducing conditions, indicating the presence of non-conformational epitopes in the S-protein but not in the RBD protein [70,71]. The sera of non-SARS-CoV-2-exposed individuals did not recognize any of the VNP-borne SARS-CoV-2 proteins (Figure 2C,D). Note that the control VNPs, expressing the major mugwort pollen allergen Art v 1 on their surface (Art v 1::GPI), were detected by the Art v 1-specific monoclonal antibody clone 5 (Figure 2E) as 40 kDa (without FLAG-tag) or 45 kDa (with FLAG-tag) bands, with the FLAG-tag-specific monoclonal antibody M2 under non-reducing conditions (Figure 2F). rArt v 1 presented as a 25 kDa band (Figure 2E). The amounts of Moloney Gag proteins were determined with the R187 antibody on the same IB after stripping; they appeared as 30 kDa bands, confirming that there were no huge differences between the control VNPs and the SARS-CoV-2 S- or RBD-expressing VNPs applied to the slots (Figure 2, bottom panels).

### 2.3. VNPs Decorated with SARS-CoV-2 Antigens Stimulate SARS-CoV-2-Specific T Cell Responses

**Figure 3:**
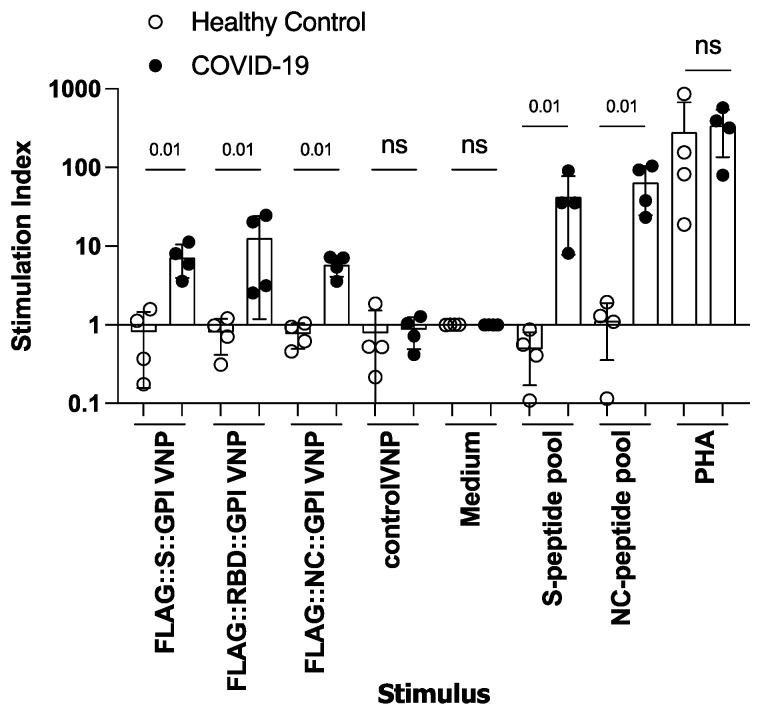
SARS-CoV-2 fusion protein-expressing VNPs induce specific T-cell proliferations in PBMC of COVID-19-convalescent but not non-SARS-CoV-2-exposed control subjects. Shown are the stimulation indices for PBMC (y-axis) of four COVID-19-convalescent individuals (closed circles) and four non-SARS-CoV-2-exposed control subjects (open circles); which were incubated with the indicated stimuli (x-axis) (VNP preparations (10 µg/mL final concentration), SARS-CoV-2 peptide mixes for S- and NC-protein (150 nM), PHA (10 µg/mL) as positive control or medium alone as negative control). Data are displayed as mean values of triplicates. Each symbol indicates PBMC of one individual. Bars indicate the means of the groups and whiskers indicate the standard deviation in the data. Average counts per minutes in medium alone were 1761 ± 1537. The *p*-values of the non-normally distributed data were calculated between the two groups for each stimulus independently of the other stimuli using the Mann–Whitney U-test; the corresponding values are shown; ns, not significant.

Next, we wondered whether T cells of convalescent patients would recognize VNP-borne SARS-CoV-2 antigens in proliferation experiments using PBMC as the responding cell population. Co-incubation of VNPs expressing the different SARS-CoV-2 proteins efficiently stimulated T lymphocytes in PBMC of COVID-19-convalescent patients but not in PBMC of non-SARS-CoV-2-exposed control subjects (Figure 3). The stimulation indices calculated between stimulated and non-stimulated samples (medium controls) amounted to 7.2 ± 3.3, 12.7 ± 11.5 and 5.8 ± 1.7 for T cells stimulated with VNPs decorated with FLAG::S::GPI, FLAG::RBD::GPI and FLAG::NC::GPI, respectively. These results suggested that the FLAG::RBD::GPI fusion proteins can also be taken up by antigen-presenting cells and presented in an immunogenic form to T cells, which subsequently leads to their significant activation. T cell stimulation by S- and NC-peptide pools (42.4 ± 34.6 and 64.6 ± 40.0) was clearly more pronounced than it was in the one induced by the virus-protein-decorated VNPs; this may, however, be a reflection of the molar concentrations of the pure peptides applied and the peptide concentrations generated by the processing of the VNP-expressed proteins. A PHA stimulation was used as a positive control and the results revealed comparable stimulation indices of 340 ± 205 and 280 ± 393 for the PBMC of both groups of individuals (mean ± SD), confirming their similar capacities to proliferate.

### 2.4. VNPs Decorated with RBD Induces a Robust Antibody Response Against Conformational Epitopes of RBD

FLAG::RBD::GPI showed the highest expression levels on producer cells (Figure 1), was well-expressed on VNP (Figure 2), induced significant T-cell proliferation (Figure 3) and since anti-RBD antibodies are important for virus neutralization; therefore, FLAG::RBD::GPI-decorated VNPs were chosen for the immunization studies to induce humoral and cellular immunity in mice.

Accordingly, we immunized mice with FLAG::RBD::GPI-decorated or non-decorated control VNPs. We have shown previously that VNPs can be productively decorated with functionally intact cytokines [20,57]. Therefore, here, we sought to evaluate whether the co-expression of IL-12::GPI on FLAG::RBD::GPI-decorated VNPs would further enhance or skew antibody production towards SARS-CoV-2. IL-12 was chosen because of its known role as an inducer of anti-viral type I immunity [61]. The VNPs used for immunizations expressed the molecules of choice, as confirmed by immunoblotting analyses (Figure 2 and Figure S1).

**Figure 4:**
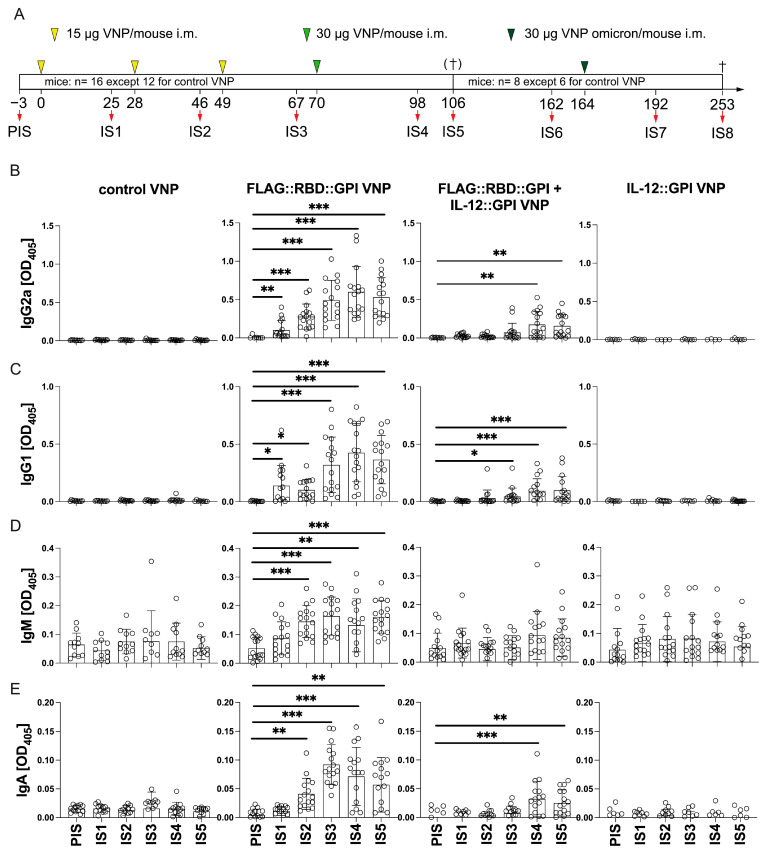
FLAG::RBD::GPI-decorated VNPs induce RBD-specific antibodies upon i.m. immunization of mice. (**A**) Shown is the scheme and numbers of mice immunized with the different VNP preparations. Yellow (15 µg) and green (30 µg) arrowheads indicate dose and time points of i.m. administration of VNP preparations. Red arrows indicate the timepoints for blood drawing. PIS, pre-immune serum; IS, immune serum. Figures (**B**–**E**) show the RBD Wuhan Hu-1-specific IgG_2a_ (**B**)**,** IgG_1_ (**C**), IgM (**D**) and IgA (**E**) reactivity (y-axes: OD_405_ values) of sera (x-axes: PIS to IS5) obtained from mice which were immunized as described on top of the Figure (FLAG::RBD::GPI, FLAG::RBD::GPI plus IL-12::GPI, IL-12::GPI decorated or non-decorated control VNPs) and analyzed by ELISA. Circles show the values of individual mice. *p*-values were calculated with the Kruskal–Wallis test following Dunn’s multiple comparison test against PIS of each group and are denoted as follows: *, *p* < 0.05; **, *p* < 0.01; ***, *p* < 0.001; only significant differences are shown.

Accordingly, BALB/c mice (*n* = 16, except 12 for control VNP) were injected four times with 15 to 30 µg of the indicated VNP preparations at 3–4-week intervals according to the schedule shown in Figure 4A. Subgroups of mice (8 of 16 mice per group, except 6 of 12 for control VNP) were boosted with a fifth immunization (day 164); in the case of the RBD-based VNPs, this now expressed the Omicron variant instead of the Wuhan Hu-1 variant (Figure 4A). Three days before each immunization serum was collected and the presence of SARS-CoV-2-specific antibodies was analyzed by ELISA. Except for some baseline IgM, the mice did not present with any significant levels of preformed SARS-CoV-2-specific antibodies in the pre-immune serum (PIS) (Figure 4B–E). RBD-specific antibody levels became only detectable in mice, which had been immunized with FLAG::RBD::GPI-expressing VNPs; this was not the case with non-decorated or IL-12::GPI-expressing control VNPs. Notably, RBD-specific IgG_2a_ and IgG_1_ levels became detectable already after the first immunization (IS1) (Figure 4B,C); from there on, they steadily increased with each further immunization until the fourth injection (IS4). Interestingly, the co-expression of IL-12::GPI on the FLAG::RBD::GPI-expressing VNPs negatively interfered with antibody development and eventually resulted in lower overall antibody levels after four immunizations, despite the expression of even higher amounts of RBD on IL-12::GPI on FLAG::RBD::GPI-expressing VNPs than on FLAG::RBD::GPI VNPs (Figures S1 and S2). In fact, after four immunizations (IS4), FLAG::RBD::GPI VNPs compared to FLAG::RBD::GPI plus IL-12::GPI VNP-immunized mice presented with significantly higher RBD-specific IgG_2a_ and IgG_1_ levels (OD_405_: 0.600 ± 0.331 and 0.425 ± 0.252 versus 0.176 ± 0.171 and 0.094 ± 0.105; *p* < 0.0001 for both comparisons). Notably, the observed antibody levels at IS4 remained relatively stable for >2 months (IS6) (Figure S2).

In contrast to IgG levels, the vaccine-induced RBD-specific IgM and IgA levels significantly increased only after the second immunization with FLAG::RBD::GPI VNPs, but not with control VNPs, FLAG::RBD::GPI VNPs co-expressing IL-12::GPI or VNPs expressing IL-12::GPI alone (Figure 4D). The only exception were the mice which were immunized at least three times with FLAG::RBD::GPI VNPs co-expressing IL-12::GPI, which also developed appreciable, albeit low, RBD-specific IgA levels (Figure 4E). IgM and IgA levels had peaked already after the third immunization (IS3) (Figure 4D,E).

**Figure 5:**
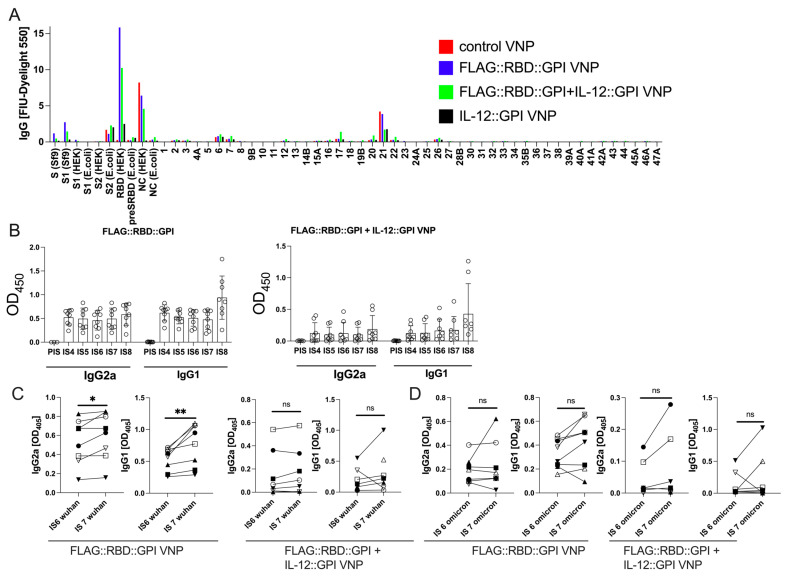
Comparison of the reactivity of mouse immune sera with overlapping peptides of SARS-CoV-2 spike protein (Wuhan Hu-1) and full-length folded or unfolded RBD Wuhan Hu-1; stability of the antibody response and effects of a single RBD–Omicron booster vaccination on the RBD Wuhan Hu-1 and RBD–Omicron-specific antibody levels. (**A**) IgG reactivity (FIU, fluorescence intensity units, Dyelight 550, y-axis) of pooled sera from 8 VNP-immunized mice, except 6 for control VNP-immunized mice, with micro-arrayed SARS-CoV-2 antigens and S-protein-derived peptides (x-axis). (**B**) Anti-RBD Wuhan Hu-1 IgG_2a_ and IgG_1_ antibody reactivity measured by ELISA (y-axes) of mice immunized with either FLAG::RBD::GPI VNPs or FLAG::RBD::GPI+IL-12::GPI VNPs from IS4 to IS8 and PIS (x-axes). (**C**,**D**) Anti-RBD-antibody reactivity of sera measured by ELISA (1:500 dilution) against coated RBD Hu-1 (**C**) or RBD–Omicron (**D**) at time points IS6 and IS7. Each mouse is represented by a different symbol. IgG_2a_ and IgG_1_ antibody levels were determined by ELISA and are depicted as OD_405_ (y-axes). *p*-values were calculated with the Kruskal–Wallis test following Dunn’s multiple comparison test (**B**) or Wilcoxon matched rank pairs test (**C**,**D**) and denoted as follows: ns, not significant, *, *p* < 0.05; **, *p* < 0.01.

Next, we analyzed the epitope specificities of the VNP-induced antibodies by ELISA and protein microarray. First, we found that immunization with FLAG::RBD::GPI-expressing VNPs induced IgG antibodies exclusively against folded but not against unfolded RBD, indicating the importance of conformational epitopes (Figure 4B–E and Figure S3). Furthermore, we applied a previously established [52] microarray containing folded and non-folded SARS-CoV-2 antigens and peptides spanning the S-protein. Notably, antibody reactivity was barely observable with any of the overlapping S-protein-derived linear peptides; however, antibody reactivity was clearly restricted to folded RBD produced in eukaryotic HEK-293T cells (Figure 5A), which resembled the binding specificity of the sera of convalescent patients, as determined previously [52]. Notably, a distinct antibody reactivity was observed with peptide 21, which represents the C-terminal sequences of the VNP-expressed RBD protein located outside the receptor binding motif. We also observed some NC-specific reactivity in the sera of mice immunized with control VNPs. This may have been caused by cross-reactive sequences/epitopes between SARS-CoV-2 NC (Protein ID.: GenBank: QHR63278.1) and MoMLV core proteins [69] (sequence identity in the alignment analyses of 65.4%); the latter is present in VNPs.

### 2.5. Primary RBD Wuhan Hu-1-Specific Antibodies Induced by VNPs Decorated with RBD Wuhan Hu-1 Can Be Boosted by Immunization with VNPs Decorated with RBD–Omicron

After four immunizations, mice maintained stable antibody levels between day 70 and day 161, i.e., for at least three months (92 days) (Figure 5B and Figure S2). On day 164, a fifth immunization, this time with RBD–Omicron-expressing VNPs, was administered in a subgroup of mice (8 of 16 mice per group, except 6 of 12 for control VNP). Notably, ELISA showed that the booster vaccination with FLAG::RBD::GPI_omicron_-expressing VNPs significantly increased the levels of RBD-specific antibodies against the RBD wildtype (Wuhan Hu-1) of the IgG2a and IgG_1_ subclasses (Figure 5C and Figure S2). While the RBD-specific antibody levels against Omicron before booster vaccination (Figure 5D) were generally lower than those directed against RBD Wuhan Hu-1 (Figure 5C), the IgG1 reactivity with RBD–Omicron in sera of mice analyzed 28 days after the Omicron booster vaccination (IS7) also increased in six out of eight (75%) mice immunized with FLAG::RBD::GPI_omicron_ VNP; however, the values did not reach statistical significance (Figure 5D). Unexpectedly, the repeated booster vaccinations with IL-12-expressing VNPs induced anti-IL-12 antibodies in mice. Such antibodies were observed in the presence and in the absence of FLAG::RBD::GPI (Figure S4).

### 2.6. RBD-Specific Antibodies Induced by VNPs Decorated with RBD Neutralize SARS-CoV-Infections in Vitro and Show ADCC Activity

**Figure 6:**
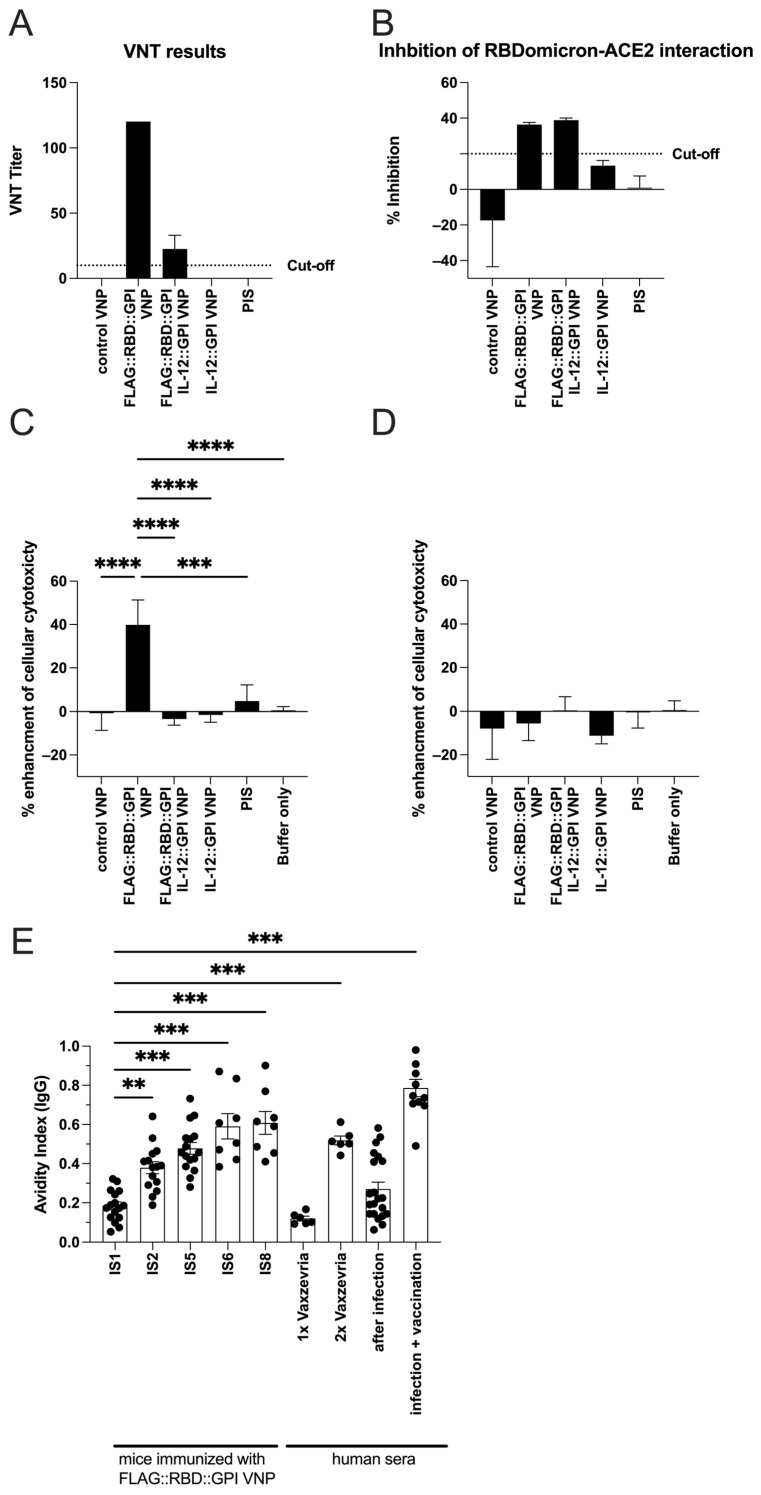
VNP-induced antibodies are functionally relevant and their avidity increases upon booster immunizations. (**A**) Virus neutralization titers (serum dilution, y-axis) of 8 mice per group, except 6 mice for control VNPs, which were tested as two independent pools consisting of 4 or 3 mice, respectively. Mice were immunized four times (IS5) with the indicated VNP preparations (x-axis). (**B**) Inhibition of RBD_omicron_ to the ACE2 binding of sera from mice immunized five times (IS8) with the indicated VNP preparations (x-axis). The dotted line indicated the cut-off of 20%. Bars indicate means and whiskers indicate standard deviations. (**C**,**D**) Enhancement of cellular cytotoxicity of LAK cells against either FLAG::RBD-expressing HEK-293T cells (**C**) or wildtype HEK-293T cells (**D**) as targets in the presence of the IS8 from mice which were immunized with the indicated VNP preparations, or in the presence of pre-immune serum (PIS) or buffer only. Bars represent the median ±95% CI enhancement of killing (y-axis). Per immunization group, sera of 8 different mice were analyzed, except 6 for mice immunized with control VNPs in 4 independent ADCC experiments, in which each serum was analyzed in duplicate over 5 different effector–target ratios (40:1, 20:1, 10:1, 5:1, 2.5:1, respectively). The cell concentrations of target cells were kept constant at 1 × 10^4^ cells per well and spontaneous lysis comprised <10% of maximum lysis in each experiment. (**E**) Avidity index (y-axis) assessed by ELISA of individual immune sera (IS) of RBD–VNP-immunized mice (x-axis) or human Vaxzevria vaccinees at the indicated timepoints for venipuncture and the history of SARS-CoV-2 infection and/or vaccination (x-axis). The results of *n* = 16 mice for IS1, 2 and 5 are shown; *n* = 8 mice for IS 6 and 8; *n* = 6 individuals 22 days after the first and 96–121 days after the second vaccination with Vaxzevria. Bars indicate means and whiskers indicate standard deviations. Every point represents the avidity index for an immunized mouse or a human subject. *p*-values were calculated with Kruskal–Wallis test following Dunn’s multiple comparison test in (**C**,**D**) and one-way ANOVA following Dunnett’s correction for multiple comparison test against IS1 of mice of each group in E and are denoted as follows: **, *p* < 0.01; ***, *p* < 0.001 and ****, *p* < 0.0001; only significant differences are shown.

Next, using virus neutralization tests, molecular interaction assays and ADCC experiments, we wanted to clarify whether the FLAG::RBD::GPI VNP-induced antibodies are also functionally relevant. Sera obtained from FLAG::RBD::GPI VNP- and FLAG::RBD::GPI+IL-12::GPI VNP-immunized mice after the fourth immunization (IS5; *n* = 8) demonstrated virus-neutralization titers (VNT) of 120 and 15–30, respectively (Figure 6A). In contrast, sera of mice immunized with control VNPs, either in the presence or absence of IL-12, were unable to neutralize viral infection (Figure 6A). In addition, sera of mice immunized with FLAG::RBD::GPI VNPs and receiving an additional FLAG::RBD::GPI_omicron_ booster vaccination, either in the presence or in the absence of IL-12::GPI, developed RBD–Omicron-blocking antibodies above the threshold of 20% (Figure 6B). However, sera of mice immunized with empty VNPs or IL-12::GPI alone did not show any blocking activity (Figure 6B). Moreover, the sera (IS8) of mice which were immunized with FLAG::RBD::GPI-decorated VNPs but not with other forms of VNPs showed a 40% enhancement (*p* < 0.0001) in antibody-dependent cellular cytotoxicity (ADCC) against HEK-293T cells expressing the SARS-CoV-2 RBD protein (Figure 6C,D).

### 2.7. Continuous Immunization with VNPs Decorated with RBD Increases Avidity of RBD-Specific Antibodies and Is Accompanied by Cytokine Responses

Next, we were interested in determining whether the sequential immunizations with FLAG::RBD::GPI VNPs had an influence on the avidity of the induced serum antibodies, as determined by ELISA. Antibody avidity was tested in the presence of 6 M urea, which had no influence on plate-bound RBD, as detected by hybrid-immune sera (Figure S5). We found a steady increase in avidity (Figure 6E), starting from a low mean avidity index of 0.18 ± 0.08 after the first (IS1), leading to a mean avidity index of 0.38 ± 0.12 (*p* = 0.0015) after the second (IS2) and culminating in a mean avidity index of 0.61 ± 0.18 (*p* < 0.0001) after the fifth immunization (IS8). For comparison, the human sera of the vaccinees were analyzed in parallel, which showed low avidity indices after one vaccination with Vaxzevria (0.12 ± 0.03) which significantly increased after two vaccinations (0.52 ± 0.06), indicating the potential usefulness of the VNP-based vaccine in comparison to established SARS-CoV-2 vaccines.

Next, we were interested in determining whether the FLAG::RBD::GPI VNPs had induced specific cytokine responses in mouse splenocytes during the proliferation assays. SARS-CoV-2 peptide-mix-induced cytokine secretion was generally moderate but was superior in the splenocytes of the FLAG::RBD::GPI VNP-immunized mice, revealing a mixed Th1/Th2 pattern (Figure S6).

## 3. Discussion

Several promising approaches for vaccination against COVID-19 based on virus-like particles expressing SARS-CoV-2 antigens have been developed, even reaching clinical trials [24,27,29]. In clinical trials, adjuvanted VNP-based vaccines have shown similar immunogenicity compared to vector- or protein-based vaccines and a slightly lower immunogenicity compared to mRNA-based vaccines with regard to the induced antibody levels, while similar cellular responses were induced by the different vaccine types [2,25,29,72,73,74]. VNP-based vaccines for SARS-CoV-2 may help to increase the number of viable options for boosting the immunity in patients who suffer from the sometimes-considerable reactogenicity that is observed after boosting with the currently available mRNA vaccines. The advantages of VNP-based vaccines are that the immunogens can be relatively easily produced in a standardized procedure and adapted for different strains. The aim of this study was to introduce *Moloney* murine leukemia virus-like nanoparticles (VNPs), which express a membrane-anchored version of the receptor-binding domain (RBD) of the SARS-CoV-2 spike protein, as platform for inducing neutralizing SARS-CoV-2-specific antibodies. Our strategy for the membrane anchoring of viral molecules took advantage of previously established technology [20,57,58,59,75] and was based on fusing the RBD domain of the SARS-CoV-2 S-protein to the minimal CD16b GPI anchor acceptor sequence. As shown previously, such VNPs proved to be stable at 4 °C for at least 4 weeks without alterations in biological activity [59]. Using this technology, we have proven that both full-length spike and nucleocapsid proteins of SARS-CoV-2 and the RBD domain of the S-protein could be successfully expressed in a folded form on HEK-293 producer cells. Already, the immunoblotting experiments performed with VNP producer cells revealed the following findings: (i) FLAG::RBD::GPI can be used to successfully decorate MoMLV-derived VNPs; (ii) the FLAG::RBD::GPI fusion protein was found to be expressed in an immunoreactive form on the surface of the VNPs, and was clearly recognized by the polyclonal serum pool of COVID-19-convalescent subjects (Figure 2A); (iii) reduction in disulfide bonds using beta-mercaptoethanol altered the conformation of FLAG::RBD::GPI in such a way that it could not be longer recognized by the serum of the COVID-19-convalescent subjects (Figure 2B); (iv) even when FLAG::RBD::GPI was expressed on the VNPs in the correct conformation (under non-reducing conditions), it was not recognized by the sera of SARS-CoV-2 non-exposed healthy control subjects (Figure 2C,D); (v) the immunoreactivity of the sera from convalescent subjects was specific in that they recognized only the VNPs expressing the FLAG::RBD::GPI fusion protein, and did not recognize the VNPs that had been decorated with other, unrelated proteins on their surface, such as FLAG::Art v 1::GPI, or that have remained undecorated (Figure 2A,B). Thus, it was tempting to speculate already at this point that the antibodies which will be induced by the vaccine will recognize conformational epitopes of RBD. The decision to focus our immunization efforts on the RBD-protein-decorated rather than the full-length S-protein-decorated VNPs was based on the analysis of sera from convalescent COVID-19 patients, which showed that antibodies binding to folded but not to unfolded RBD can block the interaction of the virus with its cellular receptor, ACE2, and thus have the potential to prevent infection [51,52].

In fact, we show that RBD-based VNPs induced RBD-specific IgG antibodies already after a single intramuscular injection even without adjuvant, which could be further augmented by additional injections. The latter also led to the production of specific other isotypes, in particular IgA, which is important for mucosal immunity, from the second injection onwards. Evaluation of the antibody binding specificities revealed that the VNP-induced antibodies were directed against conformational but not linear epitopes of RBD with virus-neutralization assays showing that the antibodies have the capacity to neutralize SARS-CoV-2 from infecting susceptible target cells. Furthermore, VNP-induced antibodies could mediate ADCC, which shows that they may also reduce the production of viruses by virus-infected cells, in addition to virus neutralization.

ELISA (Figure 4 and Figure S3) and protein array analyses (Figure 5A) revealed that RBD-based VNPs successfully induced significant levels of RBD Hu-1-specific IgG antibodies which were maintained for months. While the results obtained with protein arrays of immune sera were clear-cut, showing reactivity with folded (HEK-293 derived) but not unfolded (*E. coli*-derived) RBD, surprisingly, some antibody reactivity was also observed with peptide 21 as well as HEK-293T-produced NC (highest reactivity for mice immunized with control VNP). Those reactivities were not restricted to the sera of mice immunized with RBD-based VNPs but were also observed in the sera of mice immunized with the control VNPs; therefore, it can be assumed that they reflect cross-reactivity between a HEK-293 and/or MoMLV antigen present in the VNP preparations and peptide 21 and HEK-293 NC sequences, respectively.

Besides inducing and augmenting SARS-CoV-2 RBD-specific antibodies, we wondered whether the VNP-based vaccine promoted the avidity maturation of IgG antibodies with further shots. Primary immune responses initially generate antibodies with lower avidity, which either mature over time or mature during secondary and tertiary antigen encounters [76]. Accordingly, antibody avidity determination is a helpful tool for determining vaccine efficacy or even failure, and it can be used to discriminate between primary infections, as opposed to secondary or tertiary infections [77,78,79,80]. Indeed, here, we demonstrated that the avidity of the induced RBD-specific antibodies was moderate after the first but clearly increased with further immunizations. In addition, the avidity of the antibodies improved slightly over time, even without further immunizations, e.g., between IS5 and IS6, which can be interpreted to indicate that specific B cells not only require additional encounters with the immunogens for avidity maturation but also require sufficient time to develop their full binding capacity. In our experiments, the co-incubation of antigen–antibody reactions with 6 M urea was found to optimally destabilize low-avidity antigen–antibody interactions without removing or altering the coated antigen from the ELISA plates (Figure S5); accordingly, the remaining RBD-bound high-avidity antibodies could be reliably determined with human hybrid-immune sera.

Comparable to the natural infection and to vaccinations performed with established S Wuhan Hu-1-based vaccines, a certain cross-reactivity of the antibodies induced by RBD Wuhan Hu-1 with more recent virus variants could be expected. This was indeed the case for the antibodies induced by our RBD Wuhan Hu-1 VNP vaccine, although the antibody reactivity with RBD–Omicron compared to RBD Wuhan Hu-1 was clearly inferior, similar to the situation in human patients who were only exposed to SARS-CoV-2 Whuhan Hu-1 [81]. Along those lines, we wondered whether one additional injection, now with RBD–Omicron VNPs, would significantly increase the RBD–Omicron-specific antibody levels in mice. Indeed, this booster vaccination led to a further increase in RBD-specific antibody levels which was, however, mainly caused by an increase in preexisting RBD Wuhan Hu-1-specific antibodies (Figure 5). The latter finding speaks for the involvement of “original antigenic sin” [82], which hypothesizes that it is the preexisting antibody specificities against the original pathogen which become preferentially expanded upon encounter with a new, antigenically related pathogen, even if it displays multiple antigenic differences from the original one.

While the adaptation of the VNP platform to novel virus strains worked as initially planned and assumed by us, our hope that further decoration of RBD-based VNPs with IL-12::GPI would enhance and improve specific antibody production against surface-exposed RBD did not materialize. This was surprising, but we found that the addition of IL-12 into the vaccine construct led to the induction of IL-12-specific antibodies which likely inhibited the effect of IL-12 and thus the development of RBD-specific immunity (Figure S4). It seems that the VNP platform used herein can successfully break tolerance to self proteins such as IL-12 after repeated i.m. application, which may be useful in future studies aimed at blunting undesired cytokines response, e.g., high IL-4, IL-5 and/or IL-13 levels as observed in allergic diseases. The addition of “immune-enhancing” components has been also applied for the construction of fusion-protein-based vaccines against COVID-19. For example, RBD has been fused with the constant region of human IgG, presumably with the idea of targeting the immunogen towards antigen-presenting cells containing Fc gamma receptors [22]. It remains to be seen whether this approach will be clinically effective and free of side effects because it is quite possible that such vaccines may also induce anti-human IgG antibody responses. Based on our findings, we would therefore rather consider VNPs expressing RBD as fusion proteins with carriers which are unrelated to human proteins, as has been the case for fusion-protein-based vaccines for COVID-19 in order to utilize the principle of immunogenic carrier proteins for enhancing immunity [22].

The fact that we could detect only low levels of SARS-CoV-2-specific T cell recall responses in splenocytes of immunized mice was not entirely unexpected. It somehow mimicked the situation in human COVID-19-convalescent patients, not all of whom had developed strong T cell responses against RBD after SARS-CoV-2 infection [53,83]. Nevertheless, in our study, the regular formation of virus (RBD)-specific IgG and IgA antibodies was indicative of the critical involvement of virus-specific T cell responses providing important immunoglobulin class switch factors. In fact, for the successful class switching towards IgG_2a_, IFN-γ produced by Th1 cells is required, while the generation of IgG_1_ is favored by Th2-borne IL-4. [84] Notably, we found that, after vaccination with RBD-based VNPs, but not with control VNPs, both IFN-γ and IL-4 (among others) were clearly augmented (Figure S6).

Moreover, RBD-protein-decorated VNPs, similar to S-protein-decorated VNPs, stimulated T cells from selected COVID-19-convalescent but not from SARS-CoV-2 non-exposed individuals, which confirmed the intrinsic T cell immunogenicity of the VNP-expressed RBD.

There are some limitations in this study. BALB/c mice were used for immunization and the analyses of antibody levels. This strategy did not allow us to analyze the effect of an acute viral challenge in vivo, which is only possible in genetically modified mice or Syrian hamsters. Furthermore, the vaccine was applied i.m. to mimic the situation of most of the currently licensed human vaccines as close as possible. Other routes of application, like the sub-cutaneous or intranasal route, may be explored with the vaccine described herein, and may lead to stronger Th1 polarization, higher antibody levels and improved antibody avidity after initial vaccination. The immunizations were carried out in the absence of bona fide adjuvants, which may explain the generally lower virus-neutralization titers obtained upon vaccination with this vaccine, as compared to vaccination with other VNP-based vaccines.

## 4. Materials and Methods

### 4.1. COVID-19-Convalescent and Non-Infected Subjects

Sera and PBMC from COVID-19-convalescent individuals were obtained between 11 May 2020 and 2 July 2020 [51,85]. All subjects gave their written informed consent in accordance with the Declaration of Helsinki and as approved by the Ethics Committee of the Medical University of Vienna (EK No.: 1302/2020) on 31 March 2020. The study was conducted as a single-centered observational case–control study and was therefore not registered in an international database. For details on the study population, please refer to [51,85]. This study was supported by grants from the Austrian Science Fund, grant number: DK-W1248; the Medizinisch-Wissenschaftlicher Fonds des Bürgermeisters der Bundeshauptstadt Wien (Stiftungsfonds zur Förderung der Bekämpfung der Tuberkulose und anderer Lungenkrankheiten), grant numbers: COVID001 and COVID006; and regarding vaccine technology by a grant from the Federal State of Lower Austria, Grant: Danube Allergy Research Cluster (Danube ARC). The funders had no role in study design, data collection and analysis, decision to publish or preparation of the manuscript.

For the T-cell proliferation assays, four convalescent and four non-infected controls were randomly picked from the study cohort; for the immunofluorescence staining, five individuals with high antibody levels against SARS-CoV-2 antigens were selected.

### 4.2. Mice

Age-matched, female (6–10 weeks old), homozygous BALB/c mice were used for VNP immunization experiments according to FELASA 2014 recommendations [86].

Mouse experiments were conducted after approval by the Ethics Committee of the Medical University of Vienna and the Austrian ministry of Education, Science and Research (GZ.: 2021-0.464.314) on 26 July 2021. Litters were distributed equally to each immunization group. Mice were housed in IVC cages with environmental enrichment and water and food intake ad libitum using a 12/12 h light/dark cycle. Mice were injected intramuscularly with the indicated doses of VNP preparations (15 µg or 30 µg, as indicated, measured as total protein concentrations, in a total volume of 50 µL PBS). Sera were collected from the tail vein. Immunizations were performed in 2–4 cages per immunization group. No adverse events were observed, and no animals were excluded during the study. Mice with severe signs of pain (which were not observed) would have been eliminated from the experiment. After completion of the immunization schedule, mice were sacrificed and cellular responses in splenocytes were determined, as described in Section 4.8. The treatments of the mice were open to investigators during the experiments.

A power calculation was performed before the study to make sure that with the chosen sample size detection with a power of 80% at an effect size of 1.5 and a mean difference in ELISA titers of OD = 0.45 (STD: OD = 0.3) was possible.

### 4.3. Molecular Cloning of GPI-Anchored SARS-CoV-2 Proteins

The protein sequences of SARS-CoV-2 S-protein and RBD were taken from SARS-CoV-2 isolate WIV05, complete genome, GenBank: MN996529.1, Protein ID.: GenBank: QHR63270.2; the protein sequence of the Nucleocapsid from SARS-CoV-2 was taken from SARS-CoV-2 isolate WIV05, complete genome, GenBank: MN996529.1, Protein ID.: GenBank: QHR63278.1. For the generation of the RBD protein, amino acids 318–571 from the S-protein published under QHR63270.2, counted without leader, were used (Table S1).

All three proteins (S, RBD and NC) were fused at their N-terminus to the pre-protrypsin leader after which a 3xFLAG tag [87] and a GGGGS-linker were introduced. In the case of the S-protein, the putative transmembrane region from amino acids 1214 to 1273 was removed. At the C-terminus, the protein of interest was linked to the minimal CD16b GPI anchor acceptor sequence, taken from GenBank: X07934.1, amino acids 193–233 from GenBank: X07934.1 [58,88]. The three DNA constructs were synthesized by ATG:biosynthetics GmbH (Merzhausen, Germany) and subcloned into the expression vector pEAK12::GFP (EdgeBio, Gaithersburgh, ME, USA) by releasing the sequence of GFP by double digest with Hind III and Not I and replacing it with the above-mentioned fusion genes. The RBD–Omicron construct was synthesized by ATG:biosynthetics GmbH (Merzhausen, Germany) and subcloned directly into the expression vector pEAK12::RBD (Table S1). As controls, Art v 1::GPI [59] and a FLAG tagged version of Art v 1 GPI were used. The FLAG-tagged Art v 1 was synthesized by ATG:biosynthetics GmbH (Merzhausen, Germany) and subcloned directly into the expression vector pEAK12::RBD. A single-chain murine IL-12::GPI containing an elastin linker was subcloned from the vector pORF-mIL-12 (Invivogen, Toulouse, France) [89,90] by using the restriction sites Hind III/Nhe I (added via PCR) into a GPI::containing pEAK12 expression vector (pEAK12 CD80::GPI [20]) by removing the CD80 insert (Table S1). For VNP production, the MoMLV original gag-pol (OGP) plasmid [69] was used in addition.

### 4.4. Cell Lines and Human Primary Cells

A lab isolate of HEK-293T cells (ATCCs) was cultured and maintained in IMDM plus 10% FBS plus gentamicin (15 mg/L). PBMCs were isolated from healthy non-SARS-CoV-2-exposed individuals and COVID-19-convalescent patients. Briefly, heparinized peripheral blood was diluted 1:2 *v*/*v* in IMDM plus 10% FBS and 15 mg/L gentamicin containing 20 units /mL heparin (Gilvasan, Vienna, Austria). Ten milliliters of Ficoll in 30 mL tubes (Sterilin, Thermo Fischer Scientific, Waltham, MA, USA) were carefully overlaid with 20 mL of diluted whole blood and then centrifuged without deceleration at 500 g for 25 min. The top acellular phase was removed and the buffy coat residing on top of the Ficoll cushion containing the majority of the PBMCs was transferred into fresh 15 mL tubes using a sterile 1 mL pipette. PBMCs were washed two times with IMDM plus supplements (1st wash: 587 g, 10 min, 2nd wash: 500 g, 5 min), and were resuspended in a final volume of 1 mL and the cell concentration was determined.

### 4.5. Immunofluorescence Analyses of Producer Cells

Proper expression of SARS-CoV-2 constructs was confirmed with stable transfectants, prepared as described in [63], or with transiently transfected HEK293T cells prepared using the calcium phosphate precipitations method [64]. Briefly, 1 × 10^6^ HEK-293T cells were seeded 24 h before transfection in a Petri dish (10 cm diameter, Sarstedt) in 10 mL of IMDM plus 10% FBS plus gentamicin (15 mg/L). Two hours before transfection, medium was exchanged with 8 mL of fresh IMDM plus 10% FBS plus gentamicin (15 mg/L). For the transfection, 30 µg of pEAK12::FLAG::RBD::GPI, pEAK12::FLAG::S::GPI or pEAK12::FLAG::NC::GPI construct was diluted in 900 µL of ddH_2_O and mixed with 100 µL 2.5M CaCl_2_ solution. Afterwards, 1 mL of 2xHBS (HEPES buffered saline, 140 mM NaCl, 1.5 mM Na_2_HPO_4_, 50 mM HEPES) buffer pH 7.0 was added dropwise to the DNA solution, incubated for one minute and then added dropwise to the cells. Thus, a total of 2 mL transfection mix was added per Petri dish. Eighteen hours after transfection, the medium was replaced with 10 mL of fresh IMDM plus 10% FBS plus gentamicin and the cells were incubated for an additional 24 h. In total, 48 h after transfection, cells were harvested for flow cytometric analyses. For that purpose, cells were flushed off the plates with PBS (without Ca^2+^ and Mg^2+^) and washed twice with PBS.

Aliquots of 5 × 10^5^ cells per staining were transferred into a 4.5 mL polystyrene FACS tubes (BD) and first incubated with 0.1 µL Aqua Zombie (Biolegend, San Diego, CA, USA) at room temperature for 10 min. Afterwards, cells were washed with 4.5 mL of FACS-buffer (PBS plus 0.5% BSA and 0.05% NaN_3_), centrifuged with 500 g at 4 °C for 5 min and the supernatant was discarded. Twenty microliters of 1:100 diluted serum of either a COVID-19-convalescent or a non-infected control individual in FACS buffer or 1:200 diluted Sotrovimab (MedChemExpress LLC, Monmouth Junction, NJ, USA) were added to each tube, incubated on ice for 30 min and again washed with 4.5 mL of FACS buffer as described above. As a secondary antibody, 20 µL of 1:100 diluted goat-anti-human IgG (gamma chain specific)–APC conjugated Fab’s (Jackson Immunoresearch Laboratories, West Grove, PA, USA, Table S3) was incubated on ice for 30 min and cells were washed again afterwards. Subsequently, at least 1 × 10^4^ live cells (Aqua zombie negative singlets) were acquired on a FACS Fortessa flow cytometer (BD, Franklin Lakes, NJ, USA) equipped with the DIVA software package (BD, Version 9.0) and analyzed with the FlowJo software (Version 10.10.0).

### 4.6. Production of Virus-like Nanoparticles

For VNP (virus-like nanoparticle) production, 3 × 10^6^ HEK-293T cells were seeded onto 150 mm culture dishes, transfected the day after with 30 μg of MoMLV original gag-pol (OGP) plasmid [69] and 60 μg of pEAK12::FLAG::RBD::GPI, pEAK12::FLAG::S::GPI or pEAK12::FLAG::NC::GPI. VNP-containing supernatants were harvested after 72 h with a 25 mL pipette, centrifuged with 1000 g for 5 min to remove cellular debris, filtered (0.45 μm, Millipore, Billerica, MA), concentrated by ultrafiltration (Centricon Plus-70, Merck Millipore Ltd., Tullagreen, Ireland), and followed by further concentration by ultracentrifugation using a SW41 Ti rotor (1 × 10^5^ g, 1 h, Beckman-Optima LE-80K, Beckman Instruments, Palo Alto, CA, USA). Protein concentrations of PBS-washed VNP preparations were determined (Micro BCA, Thermo Fisher, Waltham, MA, USA) and adjusted. VNPs were stored at 4 °C until use for up to 4 weeks, without alteration of biological activity [59]. For immunization studies, 3 × 10^6^ HEK-293T cells were seeded onto 150 mm culture dishes, transfected the day after with 30 μg of MoMLV original gag-pol (OGP) plasmid and either 60 μg of pEAK12::FLAG::RBD::GPI, 60 µg of an empty control vector, a mixture of 30 μg of pEAK12::FLAG::RBD::GPI and 30 µg of pEAK12::IL-12::GPI or a mixture of 30 μg of empty control vector and 30 µg of pEAK12::IL-12::GPI. The harvesting and purification of VNPs was performed as described above. Typically, 2–6 µg of total protein of purified VNP per ml of culture volume were obtained by the described method.

### 4.7. Biochemical Analyses of Virus-like Nanoparticles

SDS–polyacrylamide gel electrophoresis (SDS-PAGE) was performed using 10 µg of purified VNP samples or 0.5 µg of recombinant Art v 1 (kindly provided by G. Gadermaier, University of Salzburg, Salzburg, Austria) and 0.25 µg of RBD_WT_ (Genescript, Piscataway, NJ, USA) per lane. The samples were mixed with a 4x SDS-PAGE sample buffer containing 40% glycerin, 200 mM Tris, 4% SDS (all from Roth, Karlsruhe, Germany) and 0.04% bromophenol blue (Sigma Aldrich, St. Louis, MO, USA), and samples were incubated for 30 min at room temperature. For reducing conditions, 5% β-mercaptoethanol was freshly added, and samples were incubated at 95 °C for 5 min. Samples were resolved on 11% polyacrylamide gels in a PerfectBLue^TM^ Vertical Dual Gel system Twin M (VWR, Radnor, PA, USA), followed by their transfer onto nitrocellulose membranes (Biorad, Hercules, CA, USA) using the electrophoretic wet blotting technique [91] in a Biorad Criterion^TM^ blotter, at 270 mA for 90 min. Subsequently, membranes were blocked with Tris-buffered saline (50 mM Tris, 150 mM NaCl, both from Roth) containing 0.05% tween-20 (Biorad) (TBS-T) and 5% non-fat dry milk (Maresi Austria, Vienna, Austria) for 1 h and afterwards incubated with a serum pool of five COVID-19-convalescent individuals with high-anti RBD levels (diluted 1:1000 in 2% non-fat dry milk in TBS-T), anti-Art v 1 mAb, clone 5 [92] (diluted 1:10 in 2% non-fat dry milk in TBS-T), anti-mIL-12 (diluted 1:1000 in 2% non-fat dry milk in TBS-T; R&D, Minneapolis, MN, USA) or anti-FLAG mAb, clone M2 (diluted 1:1000 in 2% non-fat dry milk in TBS-T; Sigma Aldrich, St. Louis, MO, USA) at 4 °C overnight. After three times washing with TBS-T and blocking of the membranes for 30 min with TBS-T containing 5% non-fat dry milk, membranes were incubated at room temperature with goat-anti-human IgG HRP-conjugated Fab’s (diluted 1:2000 in 2% non-fat dry milk in TBS-T; Jackson Immunotechnology, West Grove, PA, USA) or HRP-conjugated goat-anti-mouse immunoglobulin (diluted 1:25,000 in 2% non-fat dry milk in TBS-T; Dako, Glostrup, Denmark) for one hour. After three more washings with TBS-T and a final washing step with TBS, the blots were developed using Clarity^TM^ Western ECL substrate (Biorad, Hercules, CA, USA) as chemiluminescent indicator system and the resulting signals were captured using the LAS-4000 chemiluminescent imaging system manufactured by GE Healthcare (Chicago, IL, USA). Analyses were performed with the MultiGauge Gel Analysis Software (FUJIFILM, Minato City, Tokyo, Japan)**.**

After chemiluminescence imaging was finished, the stripping process was initiated by rinsing the membranes several times with TBS, followed by equilibration in TBS for 5 min. Stripping was performed by incubation of the membranes at room temperature 3 times for 5 min in stripping buffer (1.5% glycine, 1% Tween-20, 0.1% SDS, pH = 2.2). After the last stripping step, the membranes were extensively washed and equilibrated, as described above. Subsequently, membranes were blocked at room temperature with TBS-T containing 5% non-fat dry milk for 1 h. Afterwards, they were incubated with anti-p30gag (R187) hybridoma supernatant [93] diluted 1:20 in 2% non-fat dry milk in TBS-T, at 4° overnight. This antibody recognizes MoMLV capsid proteins thus serving as the loading control for the VNPs. After three times washing with TBS-T and blocking of the membranes with TBS-T containing 5% non-fat dry milk for 30 min, membranes were incubated at room temperature with HRP-conjugated goat-anti-rat IgG (diluted 1:2000 in 2% non-fat dry milk in TBS-T; Cytiva, Marlborough, MA, USA) for one hour. After washing three more times with TBS-T and a final washing step with TBS, the blots were developed as described above.

### 4.8. T-Cell Proliferation Assays

For the proliferation experiments of previously frozen PBMC from COVID-19-convalescent patients and non-SARS-CoV-2-exposed control subjects, 1 × 10^5^ PBMNC were incubated with purified SARS-CoV-2 molecule-decorated VNPs (10 µg/mL) (FLAG::S::GPI, FLAG::RBD::GPI, FLAG::NC::GPI), empty VNP 10 µg/mL), 150 nM of S- or NC-derived peptides (Miltenyi, Biotec, Bergisch Gladbach, Germany) media alone, or PHA (12.5 µg/mL) in 200 µL of AIM-V Medium (Thermo Fisher), plus 2% of human serum (Octapharma, Vienna, Austria) per well of round-bottom 96-well plates (Sarstedt AG, Nürmbrecht, Germany). All conditions were set up in triplicate and incubated for 144 h followed by an 18 h methyl-[3H]-thymidine pulse (1 µCi/well). After this incubation time, the cultures were harvested and T-cell proliferation was quantified on a Betaplate Counter (Perkin Elmer, Waltham, MA, USA).

For the activation of murine splenocytes, mice were sacrificed by cervical dislocation and their spleens were removed aseptically. Single cell suspensions were prepared as described previously [94]. To determine cellular factor production, 2 × 10^5^ cells were incubated per well of a round bottom 96-well plate (Sarstedt) with the different stimuli (75 µM S1 Peptide Mix, 10.4 µg/mL PHA or Medium alone) in a total of 200 µL per well of AIM-V Medium containing 1% murine serum. All conditions were set up in triplicate and incubated for 144 h followed by an 18 h methyl-[3H]-thymidine pulse (1 µCi/well). After this incubation time, T-cell proliferation was quantified on a Betaplate Counter (Perkin Elmer, Waltham, MA, USA). For cytokine analyses, supernatants were harvested after 72 h and frozen at -80 °C; they were then analyzed at a later timepoint.

### 4.9. Determination of Cytokines in Cell Culture Supernatants

Secreted mouse cytokines in the supernatant of cultured splenocytes, as described above, were analyzed as described previously by multiplexing using the Luminex platform [59]. Briefly, 5 × 10^3^ beads coated with the respective capture antibodies, (anti-mouse IL-2, IL-4, IL-5, IL-10, IL-13, IL-17A, GM-CSF, INF-γ, and TNF-α) were incubated with 30 µL aliquots of the respective cell culture supernatants in MultiscreenHTS BV 1.2 µm plates (Merck-Millipore) at 4 °C overnight. The next day, beads were washed 3 times on an automated plate washer with 100 µL/well PBS and incubated with biotinylated secondary antibodies, recognizing the respective cytokines at room temperature for one hour. After an additional washing step, samples were incubated with streptavidin-PE (all reagents from eBioscience, Thermo Fisher) at room temperature for 30 min and washed again. Finally, samples were acquired on a Luminex 100 device and the obtained fluorescence intensities of the individual bead populations were related to standard curves, obtained using cytokines with known concentrations; the absolute concentrations were calculated accordingly.

### 4.10. ELISA

Recombinant folded RBD Wuhan Hu-1 or RBD–Omicron expressed in HEK cells (Genscript, Piscataway, NJ, USA) or unfolded RBD Wuhan Hu-1 from *E. coli* [52] was coated at 2 µg/mL in a total of 40 µL in half-well plates (Greiner, Kremsmünster, Austria) in carbonated coating buffer (pH 9.6) at 4 °C overnight. The next day, the plates were washed automatically with a plate washer 3 times with 150 µL/well of washing buffer (PBS plus 0.05% Tween 20); then, 100 µL/well of blocking buffer was applied (PBS plus 3% BSA, 0.05% Tween 20) for at least 2 h at room temperature. Afterwards, 40 µL of either 1:500 diluted serum samples for IgG determination or 1:50 diluted serum samples in PBS plus 0.5% BSA, 0.05% Tween-20 for IgM and IgA determination were applied per well and incubated at 4 °C overnight. After the incubation, plates were washed as described above and 40 µL of a 1:500 diluted specific rat-anti-mouse (anti-IgM, anti-IgA, anti-IgG_1_ or anti-IgG_2_) antibody in PBS plus 0.5% BSA, 0.05% Tween 20 was applied at room temperature for 2 h. After washing, 40 µL of a 1:1500 anti-rat IgG-HRP diluted in PBS + 0.5% BSA + 0.05% Tween-20 was applied for one hour at room temperature and after washing, 100 µL of ABTS substrate was applied. OD_405_ was measured after 10–30 min on a Multiskan^TM^ GO plate reader (Thermo Scientific Fisher Inc.). All samples were measured in duplicate; non-coated, blocked wells were used as a serum-specific negative control. To calibrate the different plates, two reference samples were included on each plate.

### 4.11. Chip Analyses with Micro-Arrayed SARS-CoV-2 Antigens and Peptides

Micro-array analyses were performed as described previously [52,53]. Briefly, aliquots of SARS-CoV-2 antigens or peptides dissolved in phosphate buffer (75 mM Na_2_HPO_4_, pH 8.4) in concentrations of 0.5–1 mg/mL were spotted in triplicate onto pre-activated glass slides (Paul Marienfeld GmbH & Co. KG, Lauda-Königshofen, Germany) using a SciFlexArrayer S12 (Scienion AG, Berlin, Germany). Then, glass chips containing the microarrays were washed for 5 min with phosphate-buffered saline with 0.5% Tween-20 (PBST) and dried by centrifugation (200× *g*, 1 min) using a Sigma 2–7 centrifuge and an MTP-11113 rotor (Sigma Laborzentrifugen GmbH, Osterode am Harz, Germany). Afterwards, mouse sera were diluted 1:40 in sample diluent (Thermofisher, Waltham, MA, USA) and 35 μL of the dilutions were added per array under continuous shaking at 22 °C for 2 h. Subsequently, slides were washed and aliquots of 30 μL of 1:1000 diluted anti-mouse IgG-DyLight 550/array were applied at 22 °C for 30 min. After a final wash, slides were dried and scanned using a confocal laser scanner (Tecan, Männedorf, Switzerland). Image analysis was performed by MAPIX microarray image acquisition and analysis software (Innopsys, Carbonne, France).

### 4.12. VNT Determination

Determination of neutralizing antibody levels was performed as described previously [53]. Briefly, the SARS-CoV-2 neutralization test utilized a SARS-CoV-2 isolate obtained in Austria at the beginning of the pandemic and is based on the measurement of the cytopathic effect of the virus on cultured Vero E6 cells (ATCC CRL-1586). Diluted mouse serum pools were tested in duplicate in the assay. The 50% virus neutralization titer (VNT50) was reported as the interpolated reciprocal value of the dilution, yielding a 50% reduction in the anti-SARS-CoV-2 nucleocapsid protein staining.

### 4.13. Molecular Inhibition Assay

To determine the capacity of serum samples to inhibit RBD to ACE2 binding, a molecular interaction assay was used as described in [51,81] with the following modifications. In brief, diluted serum samples (1:4 or 1:10) were incubated for 3 h with 50 ng of His-tagged RBD–Omicron XBB.1.5 (Sino Biological, Beijing, P.R. China) followed by a 3 h overlay onto plate-bound ACE2 (2 µg/mL) (Genscript). Bound RBD was detected with a 1:2000 diluted rabbit-anti-His monoclonal antibody (Genscript) followed by incubation with a 1:2000 diluted HRP-labelled donkey-anti-rabbit IgG antibody (GE Healthcare). Detection with ABTS as substrate, measurement of the color reactions and calculation of inhibition were performed as described previously [51]. All determinations were conducted in duplicate and each result is an average of duplicate determinations with <5% difference between the individual values.

### 4.14. Determination of Antibody Avidity by ELISA

For the determination of the avidity of the different immune sera of the mice and the human individuals, an avidity ELISA according to Mohsen et al. [95] with minor modifications was applied as follows. Recombinant folded RBD (Genscript, Piscataway, NJ, USA) was coated at a concentration of 2 µg/mL in a total of 40 µL in half-well plates (Greiner, Kremsmünster, Austria) in a carbonate coating buffer (pH 9.6) at 4 °C overnight. The next day, the plates were washed with a plate washer 6 times with 150 µL/well of washing buffer (PBS plus 0.05% Tween 20); then, 150 µL/well of blocking buffer was applied (PBS plus 3% BSA, 0.05% Tween 20) at room temperature for at least 2 h. Afterwards, 40 µL per well of diluted serum samples (1:2 dilution series starting from 1:200 to 1:3200 in PBS, plus 1% BSA and 0.05% Tween-20) were applied and incubated at 4 °C overnight. After the incubation, plates were washed as described above; additionally, three incubations with either 150 µL/well PBS plus 0.05% Tween-20 containing 6 M urea or PBS plus 0.05% Tween-20 for 5 min, as a reference, were performed to dislodge antibodies with low avidity. Afterwards, plates were again washed 6 times with a plate washer with 150 µL/well of washing buffer and 40 µL of a 1:1500 diluted anti-mouse IgG-HRP (Sigma Aldrich, St. Louis, MO, USA) for mouse sera or 1:1500 diluted anti-human IgG-HRP (BD) for human sera in PBS plus 1% BSA, 0.05% Tween 20 were added and incubated at room temperature for one hour. After washing 6 times automatically with washing buffer, as described above, 40 µL of TMB substrate (Sigma Aldrich, St. Louis, MO, USA) was applied and after 10 min of incubation the reaction was stopped by addition of 40 µL 2M HCl (Roth). The OD_450_ was measured immediately after stopping on a Multiskan^TM^ GO plate reader. All samples were measured in duplicate. Immune sera w/ or w/o 6 M urea were always analyzed on the same plates. The avidity index was calculated as the area under the curve of antibody reactivity (measured as OD_450_) in the presence of 6 M urea divided by the area under the curve in the absence of 6M urea. The concentration of 6 M urea was determined to be optimal for our experiments, since it removed the low avidity antibodies from RBD but did not affect RBD bound to ELISA plates, as confirmed by the unchanged reactivity of three hybrid-immune sera.

### 4.15. Determination of Antibody-Dependent Cellular Cytotoxicity (ADCC)

In order to remove antibodies which are not specific for RBD but may react with antigens expressed on HEK-293T cells, sera were first adsorbed against HEK-293T cells as described previously [63]. For that purpose, sera were diluted 1:100 in PBS plus 0.5% BSA and 0.05% NaN_3_ and absorbed four consecutive times with HEK-293T cells. For the preparation of effector cells, lymphokine-activated killer (LAK) cells were generated as described previously [96]. Briefly, mouse splenocytes from C57B/6J were isolated according to standard protocols [94] and activated at a concentration of 2.5 × 10^6^ cells/mlL in NCM medium (RPMI-1640 plus 10% FBS, 15 mg/L gentamicin, 1x non-essential amino acids, 1 mM Sodium Pyruvate, 0.024 mM HEPES and 0.1 mM β-Mercaptoethanol) in the presence of 1000 U/mL recombinant murine IL-2 (Peprotech) for three to four days. HEK-293T wildtype cells or HEK-293T cells stably expressing FLAG::RBD::GPI [63] were used as target cells. For cytotoxicity assays, HEK-293T cells were resuspended at a concentration of 2 × 10^7^ cells/mL and labelled with an equal volume of ^51^CrNa_2_O_4_ (Perkin Elmer, Boston, MA, USA activity 1 mCi/mL) at 37 °C for 1 h. Subsequently, cells were washed four times and 1.5 × 10^5^ cells were incubated with 150 µL of the absorbed sera or buffer (PBS plus 0.5% BSA and 0.05% NaN_3_) alone at 4 °C for 1 h. After washing, 1 × 10^4^ cells were seeded into individual wells of 96-well round-bottomed tissue-culture plates and incubated with titrated numbers of LAK cells (40:1 to 2.5:1) in duplicate. Media or 2% triton-X100 were added to target cells to determine spontaneous and maximum chromium release, respectively. Subsequently, plates were centrifuged at 100 g for 5 min and incubated at 37 °C for 4 h. Afterwards, supernatants were collected with the Skatron system (Molecular Devices, Biberach an der Riss, Germany) and released radioactivity; data were determined on a Cobra II gamma-counter (Packard, Meriden, CT, USA). The percentage of specific release was determined as follows: [LAK cell induced release (cpm)—spontaneous release (cpm)]/[maximum release (cpm)—spontaneous release (cpm)] × 100. For the determination of the specific enhancement (% enhancement of cellular cytotoxicity), the specific lysis in the presence of buffer alone was subtracted at each dilution and the percentage of enhancement over buffer alone was calculated.

### 4.16. Statistical Analyzes

Data were analyzed for normal distribution. For normally distributed data, a one-way ANOVA was used to compare three or more groups; meanwhile, for non-normally distributed data, the Kruskal–Wallis test followed by Dunn’s correction was applied. To calculate differences between two non-normally distributed groups, a Mann–Whitney U-test was used. Analyzes were performed with GraphPad Prism 10 (GraphPad Software, Boston, MA, USA). Generally, *p* values below 0.05 were considered statistically significant, and these are denoted as follows *, *p* < 0.05; **, *p* < 0.01; ***, *p* < 0.001 and ****, *p* < 0.0001.

## 5. Conclusions

In conclusion, we describe a novel VNP-based SARS-CoV-2 vaccination strategy which is based on the expression of folded, GPI-anchored RBD. Our vaccine was safe and well-tolerated in mice and it was found to induce significant, long-lasting neutralizing antibody titers that are capable of exerting ADCC. The described vaccine should be easily adaptable to new antigenic variants. RBD-based VNPs induced IgG_1_, IgG_2a_ and IgA antibodies alike, the avidity of which maturated upon booster vaccinations. Thus, our VNP-based vaccination strategy may contribute to the armamentarium of COVID-19 vaccines and pandemic preparedness and responses (https://ippsecretariat.org) in the future.

## Data Availability

The data presented in this study are available on request from the corresponding author.

## Supplementary Materials

The following supporting information can be downloaded at: https://www.mdpi.com/article/10.3390/ijms26136462/s1.

## Abbreviations

ABTS	2,2′-azino-bis(3-ethylbenzothiazoline-6-sulfonic acid)
ACE-2	angiotensin-converting enzyme 2
ADCC	antibody dependent cellular cytotoxicity
BSA	bovine serum albumin
CD	cluster of differentiation
COVID-19	coronavirus disease 19
CuMV	cucumber mosaic virus
ELISA	enzyme-linked immunosorbent assay
FACS	Fluorescence-activated cell sorting
FBS	fetal bovine serum
FIU	fluorescence intensity units
GFP	green fluorescent protein
GM-CSF	granulocyte macrophage colony-stimulating factor
GPI	glycosylphosphatidylinositol
HEK	human embryonic kidney cells
HIV	human immunodeficiency virus
HRP	horseradish peroxidase
IB	immunoblot
i.m	intramuscular
IFN	interferon
Ig	immunoglobulin
IL	interleukin
IMDM	Iscove’s modified Dulbecco’s medium
IS	immune serum
ISU	ISAC standardized unit
kcpm	kilo counts per minute
LPS	lipopolysaccharide
mAb	monoclonal antibody
MoMLV	Moloney murine leukemia virus
NC	nucleocapsid protein of SARS-CoV-2
NK	natural killer
NLR	nod-like receptor
p	Protein
PBMC	peripheral blood mononuclear cells
PBS	phosphate-buffered saline
PHA	Phytohemagglutinin
pMHC	peptide MHC
RBD	receptor-binding domain
RT-PCR	reverse transcriptase polymerase chain reaction
SARS-CoV-2	severe acute respiratory syndrome coronavirus-2
SD	standard deviation
SDS-PAGE	sodium dodecyl sulfate polyacrylamide gel electrophoresis
SI	stimulation index
TBS	TRIS-buffered saline
Th	T-helper
TLR	Toll-like receptor
TMB	3, 3′,5,5′-tetramethylbenzidine
TNF	tumor necrosis factor
VLP	virus-like particles
VNP	virus-like nanoparticles
VNT	virus neutralization titer
WT	wildtype
