# Supplementary material for: Moloney Murine Leukemia Virus-like Nanoparticles Pseudo-Typed with SARS-CoV-2 RBD for Vaccination Against COVID-19
Source: Int J Mol Sci. 2025 Jul 4;26(13):6462. doi: 10.3390/ijms26136462 (PMC12250557; doi:10.3390/ijms26136462)
Supplement: Supplementary file 1 [file ijms-26-06462-s001.zip › ijms-3728424-supplementary.pdf]

Appendix S1

Online Repository

Moloney murine leukemia virus-like nanoparticles pseudo-typed with SARS-CoV-2 RBD for vaccination against COVID-19

Bernhard Kratzer[1], Pia Gattinger[2], Peter A. Tauber[1], Mirjam Schaar[1], Al Nasar Ahmed Sehgal [1], Armin Kraus[1], Doris Trapin[1], Rudolf Valenta[2, 3, 4, 5, 6*] and Winfried F. Pickl[1, 6*]

[1]Institute of Immunology, Center for Pathophysiology, Infectiology and Immunology, Medical University of Vienna, Vienna, Austria.
[2]Institute of Pathophysiology and Allergy Research, Center for Pathophysiology, Infectiology and Immunology, Medical University of Vienna, Vienna, Austria.
[3]Laboratory for Immunopathology, Department of Clinical Immunology and Allergology, I. M. Sechenov First Moscow State Medical University (Sechenov University), Moscow, Russia.
[4]LIFE Improvement by Future Technologies (LIFT) Center, Moscow, Russia.
[5]Center for Molecular Allergology, Karl Landsteiner University, Krems, Austria.
[6]Karl Landsteiner University of Health Sciences, Krems, Austria.

***) Corresponding authors:**
Winfried F. Pickl, MD
Institute of Immunology, Center for Pathophysiology, Infectiology and Immunology,
Medical University of Vienna, Lazarettgasse 19, 1090 Vienna, Austria.
Phone: (+431) 40160 33245.
Fax: (+431) 40160 933245.
Email: winfried.pickl@meduniwien.ac.at.
ORCID ID: 0000-0003-0430-4952
&
Rudolf Valenta
Department of Pathophysiology and Allergy Research
Medical University of Vienna

Waehringer Guertel 18-20
A-1090 Vienna, Austria
Tel: (+431) 40400 51080
Fax: (+431) 40400 51300
E-mail: rudolf.valenta@meduniwien.ac.at
ORCID ID: 0000-0001-5944-3365

SUPPLEMENTAL FIGURE AND TABLE LEGENDS

TABLE S1. Sequence of SARS-CoV-2 expression constructs generated and used in this study. Shown are the individual protein segments of the three SARS-CoV-2 expression constructs each consisting of, a pre-pro trypsin leader sequence, 3x FLAG-tag, a flexible linker sequence, the sequence of the protein of interest, followed by the GPI anchor acceptor sequence of CD16b. Single letter code for amino acids is used. RBD sequence within the S protein is shown in bold. Constructs were inserted with Xho I and Not I restriction sites into the pEAK 12 expression vector containing the Elongation factor1 (EF1)-alpha promoter. In addition, the sequence of the control molecule Art v 1 (FLAG::Art v 1 was designed as above described for SARS-CoV-2 proteins) and IL-12::GPI is shown.

TABLE S2. Reactivity of COVID-19 convalescent and healthy control sera with SARS-CoV-2 proteins expressed on HEK-293T cells.

The table shows the summary of three independent experiments. Serum ID A001-A005: SARS-CoV-2 non-infected controls: Serum ID B056, B115, B144-146: convalescent individuals. Convalescent individuals were selected based on high anti-SARS-CoV-2 antibody levels.

TABLE S3. Antibodies used in this study. The following binding reagents have been used either conjugated to the indicated fluorophores or enzymes or in unconjugated form. Species, isotypes, clone numbers and distributors/sources are indicated.

FIGURE S1: Analyses of VNP used for immunization experiments by immunoblotting.

Immunoblot analyses demonstrating the recognition of virus-like nanoparticles (VNP) expressing SARS-CoV-2 proteins, as well as mIL-12. Ten µg/lane of purified VNP expressing the indicated SARS-CoV-2, with and without co-expression of mIL-12, as GPI-anchored fusion proteins, VNP expressing Art v 1::GPI as control for a non-viral protein, empty control VNP, or 0.25 µg/lane of recombinant RBD_{WT}, RBD_{O} and rIL12 (mouse) or 0.5 µg/ml of recombinant Art v 1 were separated using 11% SDS-PAGE and blotted onto nitrocellulose membranes. Control VNP were generated by transfection of HEK-293T producer cells with OGP and an empty pEAK12 vector. **A)** Shown is the reactivity of a serum pool from five COVID-19 convalescent individuals with purified VNP and control proteins separated under reducing conditions (upper panels). **B)** Shown is the reactivity of anti-mIL-12 mAb. **C**) Shown is the reactivity of an anti-Art v 1 mAb (clone 5) (upper panel). Lower panels in **A-C)** show the reactivity of the anti-p30-Gag mAb R187 with MoMLV core proteins which is indicative of the presence of VNP. The position of molecular mass markers is indicated in kilodaltons (kDa). The data provided are representative of three independent experiments

FIGURE S2. Longitudinal development of anti-RBD Wuhan Hu-1 antibody levels in individual mice immunized with RBD-expressing VNP. Shown are the RBD-specific IgG_{2a} (**A, B, E, F**) or IgG_{1} (**C, D, G, H**) antibody levels measured by ELISA (y-axes) of individual mice (characterized by individual symbols), which were immunized

with FLAG::RBD::GPI VNP (**A-D**) or FLAG::RBD::GPI+IL-12::GPI VNP (**E-H**) and were either sacrificed at IS5 (**A,C,E,G**) or IS8 (**B,D,F,H**).

FIGURE S3: **RBD-specific antibodies induced in mice immunized with RBD-decorated VNP do not react with unfolded RBD Wuhan Hu-1 from *E.coli*.** Figures **A** and **B** show the RBD-specific IgG_{2a} **(A),** IgG_1 **(B)** reactivity of sera from PIS to IS5 obtained from individual mice (circles) which were immunized with FLAG::RBD::GPI, FLAG::RBD::GPI plus IL-12::GPI, IL-12::GPI or non-decorated control VNP as indicated (x-axes). The antibody levels were determined by ELISA and are shown as OD_{405} values (y-axes). **C** confirms the presence of unfolded RBD on ELISA plates by detection with an anti-His antibody but lack of reactivity with RBD-VNP immunized mouse sera (IS5). Data show reactivity in individual wells tested in two separate experiments. Shown is the reactivity of sera of n=8 VNP immunized mice per group, except 6 for control VNP immunized mice. Each serum (1:500 dilution) was measured in duplicate and corrected by blank subtraction. P-values were calculated with Kruskal-Wallis test following Dunn's multiple comparison test. Only significant differences are shown.

FIGURE S4: Mice immunized with IL-12::GPI expressing VNP develop anti-IL-12 antibodies. Figure shows the IL-12-specific IgG reactivity as OD_{405} values by ELISA of sera (y-axes) obtained from mice which were immunized with FLAG::RBD::GPI, FLAG::RBD::GPI plus IL-12::GPI, IL-12::GPI or non-decorated control VNP as indicated at PIS, IS5 and IS8 (x-axes). Shown is the reactivity of sera of n=16 VNP

immunized mice per group, except 12 for mice immunized with control VNP for PIS and IS5 and n=8 VNP immunized mice per groups, except 6 for mice immunized with control VNP for IS8. Each serum (1:100 dilution) was measured in duplicates and corrected by blank subtraction. P-values were calculated with Kruskal-Wallis test following Dunn's multiple comparison test. *, $p<0.05$; *** $p<0.001$; ****$p<0.0001$. Only significant differences are shown.

FIGURE S5: Determination of the optimal urea concentration for the avidity ELISA. Bars indicate means ± standard deviations of OD_{405} (y-axis) values of wells, coated with 2 µg/ml rRBD overnight, which were incubated 3-times for 5 minutes each time with the indicated concentrations of urea (x-axis) after blocking with PBS-T+3%BSA. The detection was performed with 3 sera of subjects, who were vaccinated and infected several times (hybrid-immunity sera). The dotted line indicates the reactivity without urea. The decrease at 7M and 8M urea indicates potential structural alterations of important epitopes on RBD for the binding of human serum.

FIGURE S6: Proliferation of splenocytes of mice immunized with FLAG::RBD::GPI expressing VNP. Shown are the stimulation indices of secreted cytokines determined in multiplex assays and obtained upon coincubation of 2 x 10^5 splenocytes of immunized mice (control VNP red, FLAG::RBD::GPI VNP blue, FLAG::RBD::GPI + IL-12::GPI green and IL-12::GPI black) sacrificed at day 253 with SARS-CoV-2 peptide mix for S-protein containing the RBD sequence and collected

after 3 days. Bars indicate the means of triplicates of splenocytes of 8 VNP immunized mice, except 6 for control immunized VNP, the whiskers the standard error of the data.

TABLE S1. Sequence of SARS-CoV-2 expression constructs generated

Protein	Amino acid sequence
preprotrypsin leader (PPT) leader	MNPLLILTFVAAALA
3 x FLAG-tag	DYKDHDGDYKDHDIDYKDDDDK
Linker	GGGGS
S protein	SQCVNLTTRTQLPPAYTNSFTRGVYYPDKVFRSSVLHSTQDLFLPFFSNVTWFHAIHVSGTNGTKRFDNPVLPFN<br>DGVYFASTEKSNIIRGWIFGTTLDSKTQSLLIVNNATNVVIKVCEFQFCNDPFLGVYYHKNNKSWMESEFRVYSS<br>ANNCTFEYVSQPFLMDLEGKQGNFKNLREFVFKNIDGYFKIYSKHTPINLVRDLPQGFSALEPLVDLPIGINITR<br>FQTLLALHRSYLTPGDSSSGWTAGAAAYYVGYLQPRTFLLKYNENGTITDAVDCALDPLSETKCTLKSFTVEKGI<br>YQTSNFRVQPTESIVRF**PNITNLCPFGEVFNATRFASVYAWNRKRISNCVADYSVLYNSASFSTFKCYGVSPTKL**<br>**NDLCFTNVYADSFVIRGDEVRQIAPGQTGKIADYNYKLPDDFTGCVIAWNSNNLDSKVGGNYNYLYRLFRKSNLK**<br>**PFERDISTEIYQAGSTPCNGVEGFNCYFPLQSYGFQPTNGVGYQPYRVVVLSFELLHAPATVCGPKKSTNLVKNK**<br>**CVNFNFNGLTGTGVLTESNKKFLPFQQFGRDIADTTDAVRDPQTLE**ILDITPCSFGGVSVITPGTNTSNQVAVLY<br>QDVNCTEVPVAIHADQLTPTWRVYSTGSNVFQTRAGCLIGAEHVNNSYECDIPIGAGICASYQTQTNSPRRARSV<br>ASQSIIAYTMSLGAENSVAYSNNSIAIPTNFTISVTTEILPVSMTKTSVDCTMYICGDSTECSNLLLQYGSFCTQ<br>LNRALTGIAVEQDKNTQEVFAQVKQIYKTPPIKDFGGFNFSQILPDPSKPSKRSFIEDLLFNKVTLADAGFIKQY<br>GDCLGDIAARDLICAQKFNGLTVLPPLLTDEMIAQYTSALLAGTITSGWTFGAGAALQIPFAMQMAYRFNGIGVT<br>QNVLYENQKLIANQFNSAIGKIQDSLSSTASALGKLQDVVNQNAQALNTLVKQLSSNFGAISSVLNDILSRLDKV<br>EAEVQIDRLITGRLQSLQTYVTQQLIRAAEIRASANLAATKMSECVLGQSKRVDFCGKGYHLMSFPQSAPHGVVF<br>LHVTYVPAQEKNFTTAPAICHDGKAHFPREGVFVSNGTHWFVTQRNFYEPQIITTDNTFVSGNCDVVIGIVNNTV<br>YDPLQPELDSFKEELDKYFKNHTSPDVDLGDISGINASVVNIQKEIDRLNEVAKNLNESLIDLQELGKYEQYIKW<br>P
RBD protein	PNITNLCPFGEVFNATRFASVYAWNRKRISNCVADYSVLYNSASFSTFKCYGVSPTKLNDLCFTNVYADSFVIRG<br>DEVRQIAPGQTGKIADYNYKLPDDFTGCVIAWNSNNLDSKVGGNYNYLYRLFRKSNLKPFERDISTEIYQAGSTP<br>CNGVEGFNCYFPLQSYGFQPTNGVGYQPYRVVVLSFELLHAPATVCGPKKSTNLVKNKCVNFNFNGLTGTGVLTE<br>SNKKFLPFQQFGRDIADTTDAVRDPQTLE
RBD omicron	PNITNLCPFDEVFNATRFASVYAWNRKRISNCVADYSVLYNLAPFFTFKCYGVSPTKLNDLCFTNVYADSFVIRG<br>DEVRQIAPGQTGNIADYNYKLPDDFTGCVIAWNSNKLDSKVSGNYNYLYRLFRKSNLKPFERDISTEIYQAGNKP<br>CNGVAGFNCYFPLRSYSFRPTYGVGHQPYRVVVLSFELLHAPATVCGPKKSTNLVKNKCVNFNFNGLKGTGVLTE<br>SNKKFLPFQQFGRDIADTTDAVRDPQTLE
NC protein	MSDNGPQNQRNAPRITFGGPSDSTGSNQNGERSGARSKQRRPQGLPNNTASWFTALTQHGKEDLKFPRGQGVPIN<br>TNSSPDDQIGYYRRATRRIRGGDGKMKDLSPRWYFYYLGTGPEAGLPYGANKDGIIWVATEGALNTPKDHIGTRN<br>PANNAAIVLQLPQGTTLPKGFYAEGSRGGSQASSRSSSRSRNSSRNSTPGSSRGTSPARMAGNGGDAALALLLLD<br>RLNQLESKMSGKGQQQQGQTVTKKSAAEASKKPRQKRTATKAYNVTQAFGRRGPEQTQGNFGDQELIRQGTDYKH<br>WPQIAQFAPSASAFFGMSRIGMEVTPSGTWLTYTGAIKLDDKDPNFKDQVILLNKHIDAYKTFPPTEPKKDKKKK<br>ADETQALPQRQKKQQTVTLLPAADLDDFSKQLQQSMSSADSTQA
GPI-anchor acceptor sequence of CD16b	SLAVSTISSFSPPGYQVSFCLVMVLLFAVDTGLYFSVKTNI
Art v 1	AGSKLCEKTSKTYSGKCDNKKCDKKCIEWEKAQHGACHKREAGKESCFCYFDCSKSPPGATPAPPGAAPPPAAGG<br>SPSPPADGGSPPPPADGGSPPVDGGSPPPPSTHASSLAVSTISSFSPPGYQVSFCLVMVLLFAVDTGLYFSVKTN<br>I
mIL-12::GPI	MGQSRYLLFLATLALLNHLSLARVIPVSGPARCLSQSRNLLKTTDDMVKTAREKLKHYSCTAEDIDHEDITRDQT<br>STLKTCLPLELHKNESCLATRETSSTTRGSCLPPQKTSLMMTLCLGSIYEDLKMYQTEFQAINAALQNHNHQQII<br>LDKGMLVAIDELMQSLNHNGETLRQKPPVGEADPYRVKMKLCILLHAFSTRVVTINRVMGYLSSAVPGVGVPGVG<br>GSMWELEKDVYYVEVDWTPDAPGETVNLTCDTPEEDDITWTSDQRHGVIGSGKTLTITVKEFLDAGQYTCHKGGE<br>TLSHSHLLLHKKENGIWSTEILKNFKNKTFLKCEAPNYSGRFTCSWLVQRNMDLKFNIKSSSSPPDSRAVTCGMA<br>SLSAEKVTLDQRDYEKYSVSCQEDVTCPTAEETLPIELALEARQQNKYENYSTSFFIRDIIKPDPPKNLQMKPLK<br>NSQVEVSWEYPDSWSTPHSYFSLKFFVRIQRKKEKMKETEEGCNQKGAFLVEKTSTEVQCKGGNVCVQAQDRYYN<br>SSCSKWACVPCRVRSGGGASLAVSTISSFSPPGYQVSFCL

TABLE S2. Reactivity of sera from COVID-19 convalescent and non-SARS-CoV-2 exposed control subjects with the SARS-CoV-2 proteins expressed on HEK-293T producer cells

		Expressed SARS-CoV-2 proteins			
Serum ID	anti-human IgG APC	none (empty vector)	FLAG::S::GPI	FLAG::RBD::GPI	FLAG::NC::GPI
Non-SARS-CoV-2 exposed					
A001*	+	47.6±15.0	30.7±7.3	32.9±11.5	43.7±23.1
A002	+	13.8±2.2	18.0±2.8	25.6±4.8	25.8±4.7
A003	+	15.8±1.8	21.8±3.0	24.6±1.0	37.7±17.9
A004	+	20.5±2.9	23.2±3.0	24.8±1.5	29.1±5.4
A005	+	14.6±1.8	19.4±2.1	17.4±1.2	24.8±2.8
**mean reactivity±SEM**		**22.4±4.4**	**22.6±8.6**	**25.5±2.5**	**31.6±21.9**
COVID-19 convalescent					
B056	+	16.3±1.1	245.8±63	654.0±288.	154.6±35.6
B115	+	19.5±3.0	164.1±33.7	150.3±30.5	41.0±6.3
B144	+	18.4±0.5	441.3±113.4	472.0±131.0	122.9±23.5
B145	+	23.7±3.1	193.3±65.2	407.5±103.3	81.2±11.4
B146	+	15.9±2.6	95.0±16.3	105.9±36..9	31.3±3.6
**mean reactivity±SEM**		**40.0±15.3**	**227.9±37.8**	**371.2±78.8**	**86.2±13.4**
no serum	+	19.3±12.8	30.7±36.2	32.9±9.1	22.5±16.1
no serum	-	12.6±5.2	24.8±7.2	12.7±2.5	26.5±8.3

Table shows the summary of three independent experiments. Serum ID A001-A005: SARS-CoV-2 non-infected controls, B056, B115, B144-146: convalescent individuals;

TABLE S3. List of antibodies used within this study.

Specificity	Clone Name	Species	Conjugated to	Source	Cat. No.
**Flow cytometry**					
Human IgG	Polyclonal	Goat	APC	Jackson Immuno	109-136-098
Human IgG	Polyclonal	Goat	PE	Jackson Immuno	109-116-170
RBD	Sotrovimab VIR 7831	Human	-	MedChemExpress LLC	HY-P99340
FLAG	L5	Rat IgG_{2a}	PE	Biolegend	637310
**ELISA and CHIP analyses**					
Mouse IgG2a	R19-15	Rat	unconjugated	BD	553387
Mouse IgG1	A85-1	Rat	unconjugated	BD	553440
Mouse IgM	II/41	Rat	unconjugated	BD	5553435
Mouse IgA	C10-3	Rat	unconjugated	BD	556969
Rat IgG	Polyclonal	Goat	HRP	Cytiva	NA93V
Anti-Mouse IgG	Polyclonal	Goat	Dylight550	Thermo Scientific	84540
Anti Human IgG	G18-145	Mouse	HRP	BD	555788
Anti Mouse IgG	Polyclonal	Sheep	HRP	Cytiva	NA931V
His	25B6E11	Rabbit	-	Genscript	A01857
Rabbit	Polyclonal	donkey	HRP	Cytiva	NA934
**Immunoblotting**					

Human IgG	Polyclonal	Mouse	HRP	BD	5557888
Human IgG	Polyclonal	Goat	HRP	Jackson Immuno	109-036-008
P30gag	R187	Rat	unconjugated	ATCC (CRL-1912)	produced in house
Rat IgG1	Polyclonal	Goat	HRP	Cytiva	NA93V
Art v 1	Clone 5	Mouse	unconjugated	Bauer et al. [62]	produced in house
Mouse Ig	Polyclonal	Goat	HRP	Dako	P044701-2
Mouse IL-12	Polyclonal	Goat	unconjugated	R&D	AB-419-NA
Goat Ig	Polyclonal	Rabbit	HRP	Dako	P0160
Anti-FLAG	M2	Mouse	unconjugated	Sigma Aldrich	F3165

Table shows the specificities, clone names, species and suppliers (sources) of the respective antibodies and their conjugation with the respective fluorophores or enzymes used in this study.

FIGURE S1

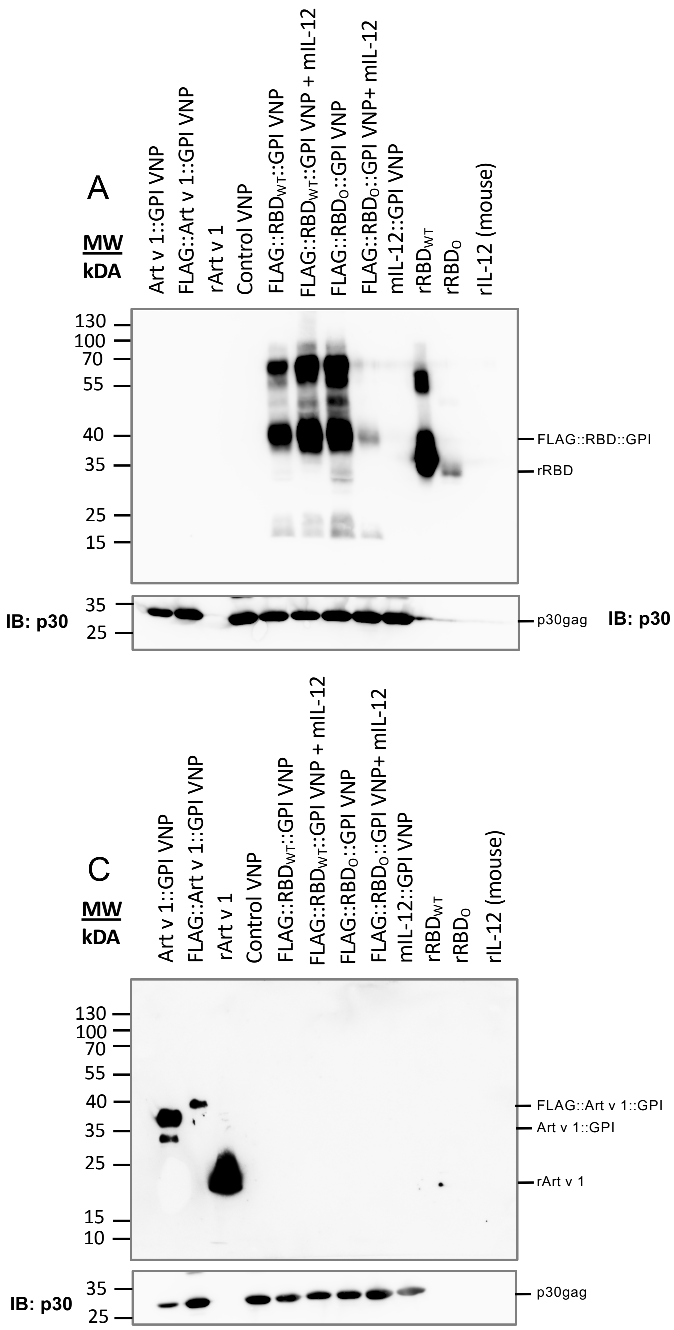
A
MW kDA
Art v 1::GPI VNP
FLAG::Art v 1::GPI VNP
rArt v 1
Control VNP
FLAG::RBD$_{WT}$::GPI VNP
FLAG::RBD$_{WT}$::GPI VNP + mIL-12
FLAG::RBD$_{O}$::GPI VNP
FLAG::RBD$_{O}$::GPI VNP+ mIL-12
mIL-12::GPI VNP
rRBD$_{WT}$
rRBD$_{O}$
rIL-12 (mouse)
130
100
70
55
40
35
25
15
FLAG::RBD::GPI
rRBD
IB: p30
35
25
p30gag
B
MW kDA
Art v 1::GPI NP
FLAG::Art v 1: :GPI VNP
rArt v 1
Control VNP
FLAG::RBD$_{WT}$::GPI VNP
FLAG::RBD$_{WT}$::GPI VNP + mIL-12
FLAG::RBD$_{O}$::GPI VNP
FLAG::RBD$_{O}$::GPI VNP+ mIL-12
mIL-12::GPI VNP
rRBD$_{WT}$
rRBD$_{O}$
rIL-12 (mouse)
130
100
70
55
40
35
25
15
IL-12::GPI
rIL-12
IB: p30
35
25
p30gag
C
MW kDA
Art v 1::GPI VNP
FLAG::Art v 1::GPI VNP
rArt v 1
Control VNP
FLAG::RBD$_{WT}$::GPI VNP
FLAG::RBD$_{WT}$::GPI VNP + mIL-12
FLAG::RBD$_{O}$::GPI VNP
FLAG::RBD$_{O}$::GPI VNP+ mIL-12
mIL-12::GPI VNP
rRBD$_{WT}$
rRBD$_{O}$
rIL-12 (mouse)
130
100
70
55
40
35
25
15
10
FLAG::Art v 1::GPI
Art v 1::GPI
rArt v 1
IB: p30
35
25
p30gag

FIGURE S2

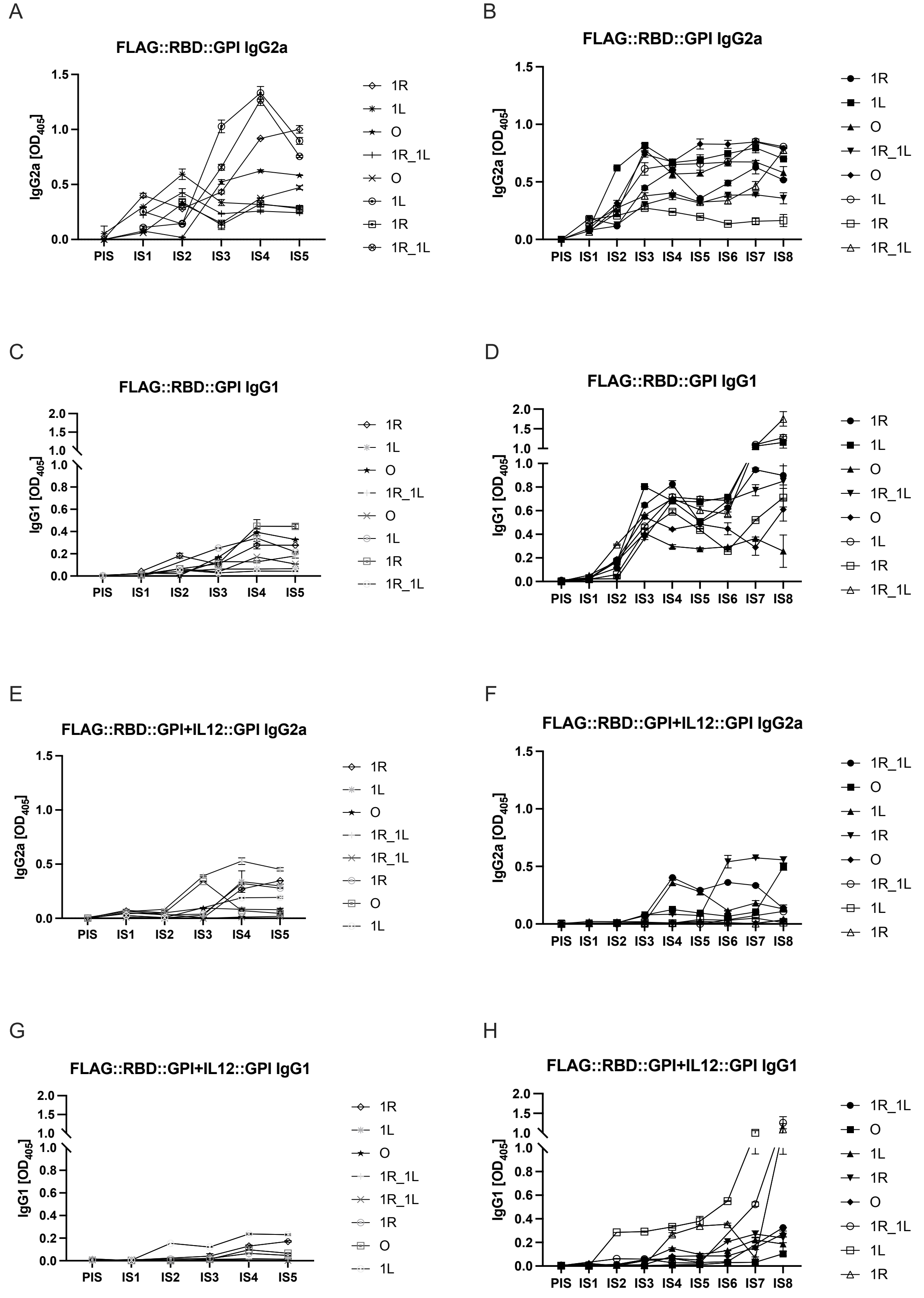
A
FLAG::RBD::GPI IgG2a
IgG2a [OD405]
PIS IS1 IS2 IS3 IS4 IS5
1R
1L
O
1R_1L
O
1L
1R
1R_1L
B
FLAG::RBD::GPI IgG2a
IgG2a [OD405]
PIS IS1 IS2 IS3 IS4 IS5 IS6 IS7 IS8
1R
1L
O
1R_1L
O
1L
1R
1R_1L
C
FLAG::RBD::GPI IgG1
IgG1 [OD405]
PIS IS1 IS2 IS3 IS4 IS5
1R
1L
O
1R_1L
O
1L
1R
1R_1L
D
FLAG::RBD::GPI IgG1
IgG1 [OD405]
PIS IS1 IS2 IS3 IS4 IS5 IS6 IS7 IS8
1R
1L
O
1R_1L
O
1L
1R
1R_1L
E
FLAG::RBD::GPI+IL12::GPI IgG2a
IgG2a [OD405]
PIS IS1 IS2 IS3 IS4 IS5
1R
1L
O
1R_1L
1R_1L
1R
O
1L
F
FLAG::RBD::GPI+IL12::GPI IgG2a
IgG2a [OD405]
PIS IS1 IS2 IS3 IS4 IS5 IS6 IS7 IS8
1R_1L
O
1L
1R
O
1R_1L
1L
1R
G
FLAG::RBD::GPI+IL12::GPI IgG1
IgG1 [OD405]
PIS IS1 IS2 IS3 IS4 IS5
1R
1L
O
1R_1L
1R_1L
1R
O
1L
H
FLAG::RBD::GPI+IL12::GPI IgG1
IgG1 [OD405]
PIS IS1 IS2 IS3 IS4 IS5 IS6 IS7 IS8
1R_1L
O
1L
1R
O
1R_1L
1L
1R

FIGURE S3

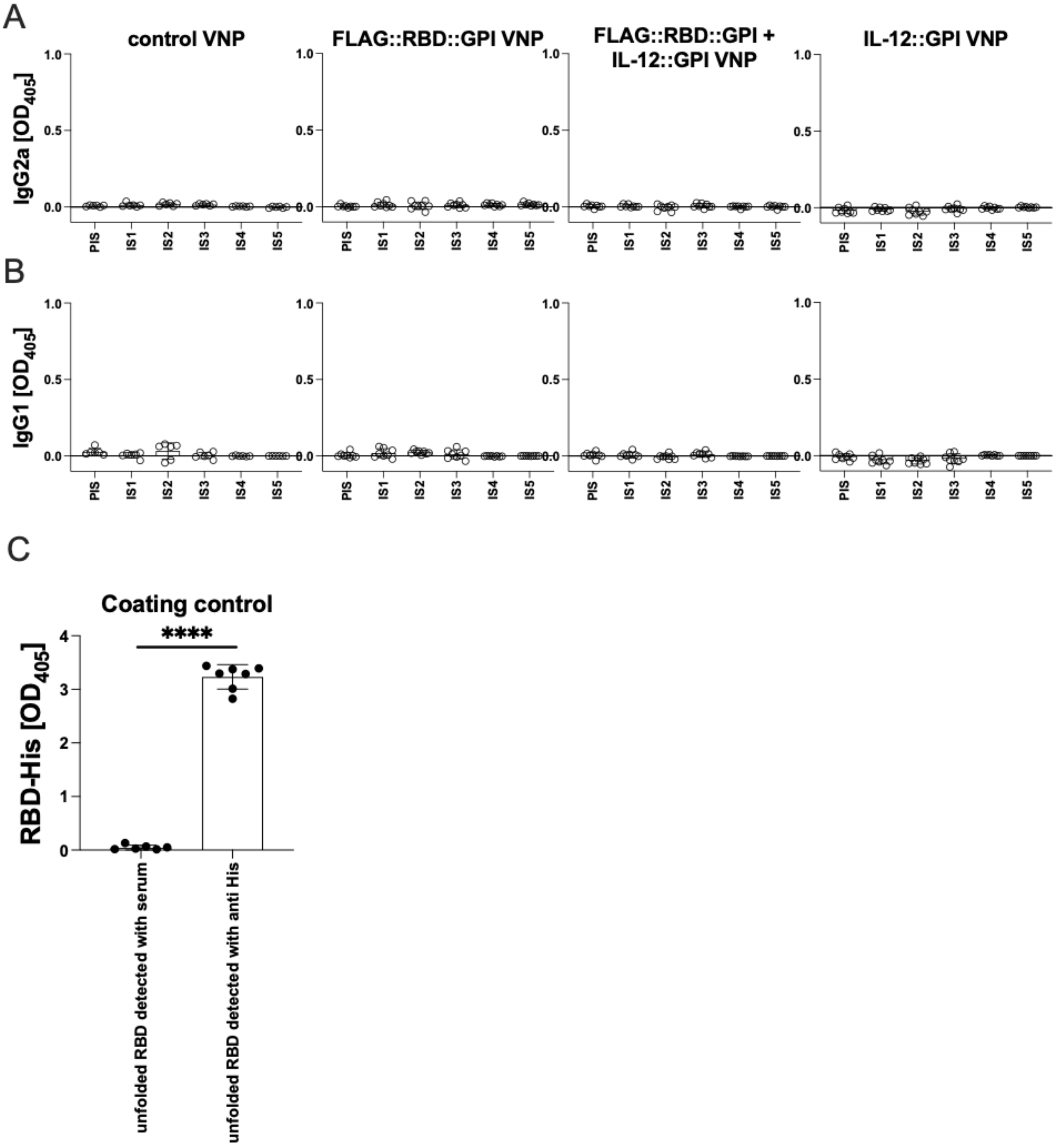
A
control VNP
FLAG::RBD::GPI VNP
FLAG::RBD::GPI +
IL-12::GPI VNP
IL-12::GPI VNP
IgG2a [OD405]
1.0
0.5
0.0
PIS
IS1
IS2
IS3
IS4
IS5
B
IgG1 [OD405]
C
Coating control
****
RBD-His [OD405]
4
3
2
1
0
unfolded RBD detected with serum
unfolded RBD detected with anti His

FIGURE S4

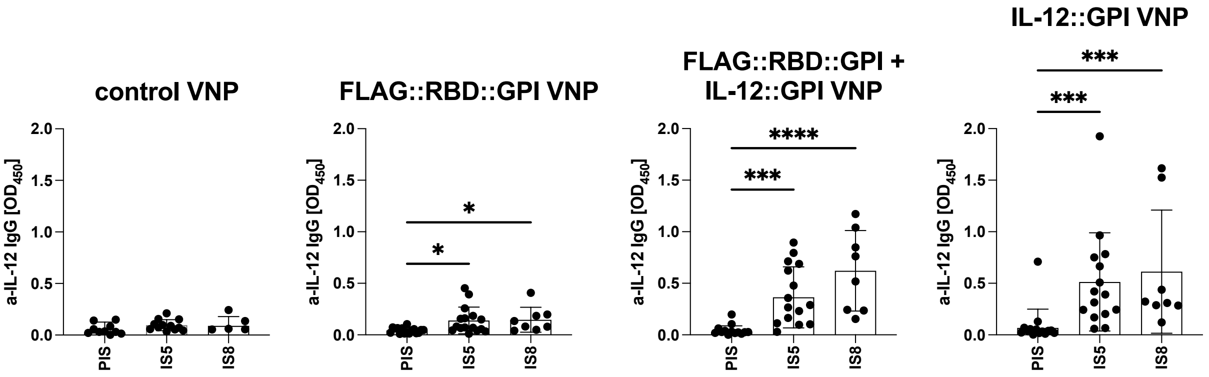
control VNP
FLAG::RBD::GPI VNP
FLAG::RBD::GPI +
IL-12::GPI VNP
IL-12::GPI VNP
a-IL-12 IgG [OD450]
2.0
1.5
1.0
0.5
0.0
PIS
IS5
IS8
*
***
****

FIGURE S5

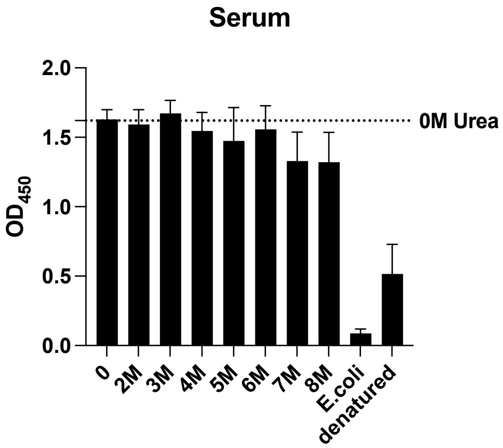
Serum
OD450
2.0
1.5
1.0
0.5
0.0
0M Urea
0
2M
3M
4M
5M
6M
7M
8M
E.coli
denatured

FIGURE S6

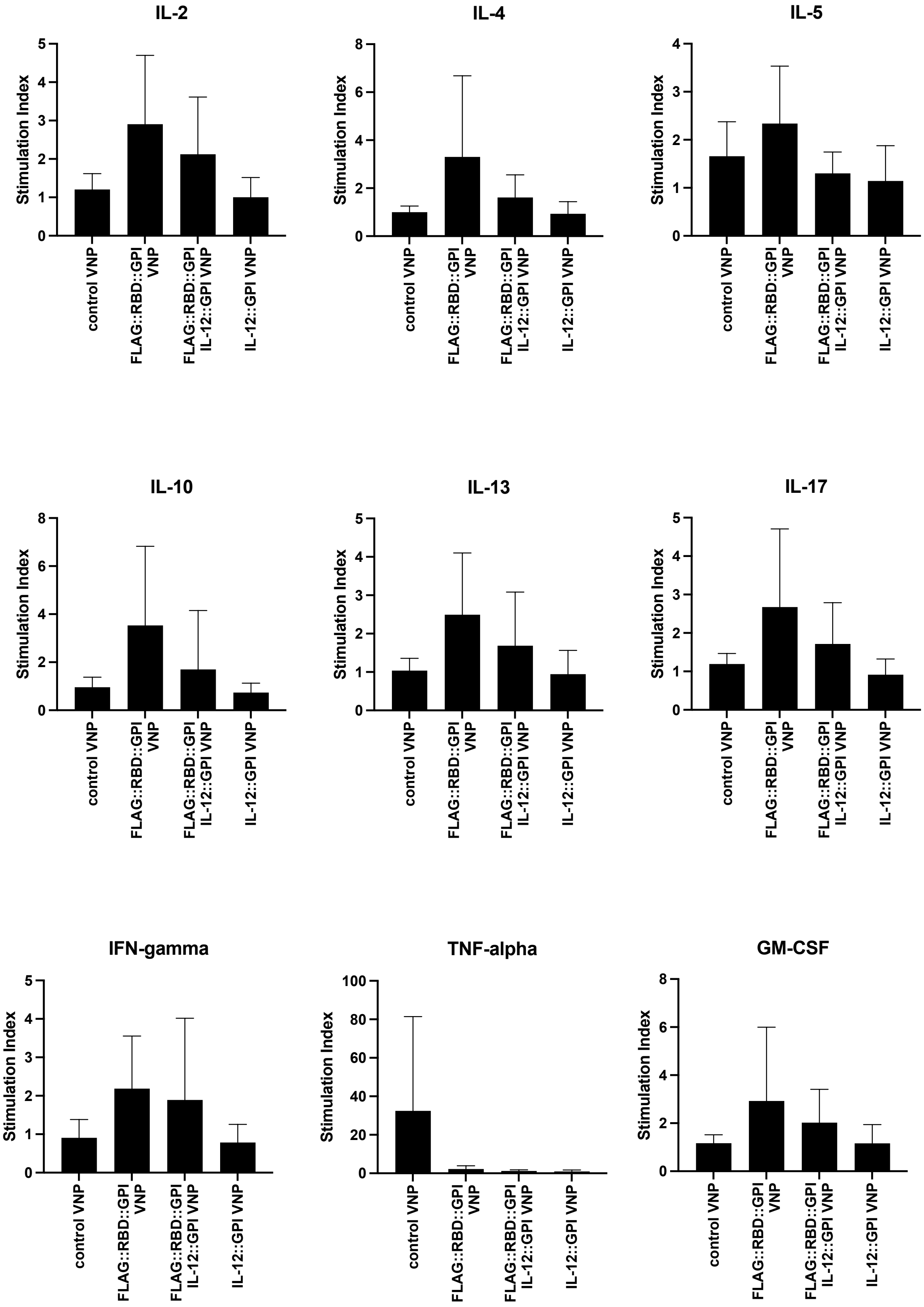
IL-2
IL-4
IL-5
IL-10
IL-13
IL-17
IFN-gamma
TNF-alpha
GM-CSF
Stimulation Index
control VNP
FLAG::RBD::GPI VNP
FLAG::RBD::GPI IL-12::GPI VNP
IL-12::GPI VNP
